# Advancing a “Good Life” for Farm Animals: Development of Resource Tier Frameworks for On-Farm Assessment of Positive Welfare for Beef Cattle, Broiler Chicken and Pigs

**DOI:** 10.3390/ani12050565

**Published:** 2022-02-23

**Authors:** Elizabeth Rowe, Siobhan Mullan

**Affiliations:** 1 Bristol Veterinary School, Langford House, Dolberry, Bristol BS40 5DU, UK; 2 School of Veterinary Medicine, University College Dublin, Belfield, D04 V1W8 Dublin, Ireland; siobhan.mullan@ucd.ie

**Keywords:** animal welfare, positive welfare, good life, farm assurance scheme, chicken, beef cattle, pig, resource needs

## Abstract

**Simple Summary:**

A “good life” for farmed animals is a life where positive experiences (positive welfare) far outweigh any negative experiences (negative welfare). If we give animals resources that they value and that provide them with positive physical and mental experiences, we give them opportunities to experience positive welfare and therefore a good life. Evaluating whether certain resources are provided for animals gives us a practical way of assessing positive welfare on farms. We describe the initial development of such resource evaluation frameworks (“Good Life Frameworks”) for beef cattle, broiler chickens and pigs.

**Abstract:**

There is increasing recognition that farm animal welfare standards should ensure positive welfare, as well as prevent negative welfare. Resources that are valued by an animal and that provide opportunities to engage in motivated behaviours can elicit positive physical and emotional states and therefore positive welfare and a “good life” for farmed animals. Evaluation of resource provision is considered the best way of assessing positive welfare at present, in the absence of validated and practical animal-based measures. Previous research has outlined a framework of three tiers of increasingly positive welfare (Welfare +, Welfare ++, Welfare +++) containing resources that incrementally increase the opportunities for a good life over and above the requirements of UK law and code of practice. Based on this blueprint, “Good Life Frameworks” were developed for beef cattle, broiler chickens and pigs, containing resources that increase good life opportunities according to the scientific literature and expert consultation. We describe the initial development of these frameworks, including a piloting exercise with the UK farm assurance industry, to further refine the frameworks according to auditor and farmer feedback, and test the frameworks as a method of on-farm assessment and assurance of a “good life” for farm animals.

## 1. Introduction

Ensuring good animal welfare means not only protecting animals from negative experiences such as pain and suffering but also ensuring that they enjoy positive experiences such as comfort and pleasure [1]. Positive welfare is a facet of animal welfare that encompasses the positive aspects of an animal’s life, i.e., positive physical and mental experiences. Although there is no universally agreed definition of positive welfare, a review of the literature suggested that positive welfare includes four key defining features—positive emotions, positive affective engagement, good quality of life and happiness [2]. 

Positive welfare is becoming an increasing focus of animal welfare science [3,4]. The reasons behind this are thought to be recognition that (1) most of the focus in animal welfare research and policy thus far has been on preventing negative welfare; (2) the absence of negative welfare (i.e., preventing suffering) is not the same as the presence of positive welfare (i.e., having positive physical and mental experiences); (3) non-human animals are capable of experiencing positive feelings, based on evidence from neuroscience and behavioural science; (4) there may be wider benefits linked to positive welfare, e.g., positive feelings may promote health; and (5) the general public associate animal welfare with the provision of opportunities for positive experiences, believing that preventing suffering is a baseline rather than the main component of animal welfare, according to social science research [4].

Yeates and Main [3] consider that a focus on positive welfare is also beneficial, beyond improving an animal’s life, for a number of reasons: (1) Society may directly value an increase in positive welfare; (2) enriching an animal’s welfare can also enrich the carer’s welfare and (3) rewarding positive outcomes may motivate animal carers more than penalising poor performance, whilst achieving the same goal of avoiding negative welfare. As a baseline, animals should be free from suffering and have all their basic needs met. However, as Yeates and Main [3] state, “what use is there in satisfying an animal’s vital needs, if the life the animal then lives is devoid of any enjoyment?”.

The question then is, how do we achieve positive welfare for the animals in our care – how do we bring enjoyment into their lives? Positive emotions are thought to arise when animals are given the opportunity to perform behaviours that they are motivated to perform. Performing such behaviours produces a positive mental state, or affect, that acts as a reward, and as such, reinforces the behaviour. This is known as “positive affective engagement”, described as “the experience animals may have when they actively respond to motivations to engage in rewarding behaviours, and it incorporates all associated affects that are positive” [5]. This theory provides a functional link between behaviours such as foraging, maternal care and play, and positive subjective experiences [4].

Following from this theory, providing resources that give animals opportunities to engage in motivated behaviours will elicit positive affect and therefore positive welfare. In addition, certain resources will give animals opportunities to experience positive physical experiences, such as physical and thermal comfort, feeling well-rested, satiated, etc. Giving animals such opportunities has underpinned the UK Farm Animal Welfare Committee’s (FAWC) recommended approach to provide animals with a “good life”, defined as a life where positive welfare substantially outweighs negative welfare and where all of the animals’ needs and most of the animals’ wants are met, a standard of welfare substantially higher than what the implementation of current UK legal minimum requirements can achieve [6]. The Committee set out four categories of a good life: Comfort, pleasure, interest and confidence. They defined these categories as “good life opportunities”. According to FAWC, “an opportunity that would be considered to contribute to a good life would be a resource that an animal does not need for biological fitness but is valued (i.e., used) by the animal” [6]. FAWC did not provide any specific resources for different good-life opportunities for any species, beyond giving a generic example for each of the four opportunities (comfort: A range of (comfortable) environmental temperatures within an animal house; pleasure: Diet with varied constituents; interest: Novel objects provided for inquisitive animals; confidence: Housing design that enables intermittent avoidance of other animals or people).

The good-life opportunities approach was further developed by Edgar et al. [7], who determined the resources needed by laying hens (*Gallus gallus domesticus*) to experience each of FAWC’s good-life opportunities, using scientific literature, expert opinion and on-farm piloting. The authors created a “Good Life Framework” of three tiers of increasingly positive welfare (Welfare +, Welfare ++, Welfare +++) containing resources that incrementally increased the opportunities for a good life over and above the requirements of UK law and code of practice. Based on Dawkins’ definition of animal welfare as “is the animal healthy, and does it have what it wants?” [8], Edgar et al. [7] added a fifth good-life opportunity of a “healthy life”.

The Good Life Framework [7] also included the concept of choice, a major theme in the wider animal welfare literature. Giving animals choices in preference tests enables us to determine the things that they “want” [8]. What animals want can mean what they want as a species and what they want as individuals. Therefore, as well as providing animals with resources we know that, as a species, they are motivated to obtain, we should also retain some choice in their environment, allowing for individual variation in preferences. In support of this, FAWC proposed that the requirements for a good life should include the “availability of environmental choices” [6]. Giving animals choice also enables them to exert a certain level of control over their lives, i.e., choosing to some extent when, where and what to eat, who to spend time with, where and on what to rest, etc. Control (along with predictability) in life and giving animals a sense of agency are considered important factors for increasing animal welfare [9] and increasing the opportunities for animals to engage in behaviours they find rewarding [10], therefore contributing to positive welfare.

The Good Life Framework is based on input (resource based) measures. Although outcome (animal based) measures of animal welfare are preferable to input measures in that they provide direct measurements of welfare at the animal level, they are more difficult to standardise and record, and animal-based indicators of positive welfare are yet to be validated [4] (i.e., ensuring that the indicators accurately reflect welfare by establishing a correlation with known physiological and/or behavioural indicators of welfare). For example, the Welfare Quality^®^ protocol, an animal welfare assessment system using animal-based measures, includes the criterion “positive emotional state” measured via qualitative behavioural assessment (QBA) [11]. QBA involves observing the animals’ body language and scoring a number of behavioural descriptors on a continuous scale. However, QBA has not been validated as a welfare measure in all farm species, and a recent study with laying hens found no associations between QBA scores and feather cover, fearfulness or mortality [12], which are well-validated measures of hen welfare [13]. Likewise, the animal-based indicators of ear position, play, allogrooming, brush use and QBA have been proposed as indicators in a prototype positive welfare assessment protocol for cattle, but this protocol has not been tested in practice and is “very far from a fully validated positive welfare protocol in an ideal world” [14].

Indeed, a review of the literature suggests that the best way of assessing positive welfare at present is by evaluation of resources (i.e., inputs) that are valued by an animal [4]. The effect of input measures on the positive welfare state of animals does need to be verified by positive outcomes such as behavioural responses, influences on cognitive processes and physiological markers [3]. However, literature on the verification of positive welfare remains lacking; we are yet to find the ‘gold standard’ for assessing and validating emotional states in animals [4]. Of the tools we do have available, e.g., cognitive bias tests [15], these are time-consuming and impractical to carry out in commercial settings with farm animals. 

Thus, a resource-based approach is the best starting point we have on our journey to ensuring that all animals in our care have the opportunity to live a good life, and the Good Life Framework provides a resource-based assessment method for this reason. 

The only other notable framework for animal welfare assessment that incorporates positive welfare other than the Good Life Framework is the Five Domains Model [16]. Domains 1 to 3 of this model (nutrition, environment and health) focus on resource-based measures of welfare, whereas Domain 4 (behavioural interactions) is intended to capture animal-based behavioural outputs, all of which affect Domain 5 (mental state). However, the model does not detail the behavioural indicators necessary for the model’s utilisation as a practical on-farm assessment tool. Indeed, a suggestion for translating the Five Domains theoretical model into a framework for on-farm assessment of animal welfare enhancement is based directly on the Good Life Framework [10]. Furthermore, the “welfare enhancing affects” of comfort, pleasure, interest, attachment, confidence and a sense of being in control described in the Five Domains model [16] directly correspond to the FAWC good-life opportunities and the provision of choice to enable control used in the Good Life Framework. Opportunities identified for increasing positive welfare in pigs using the Five Domains model involved providing resources for pigs (environmental enrichment and positive human–animal interactions) [17], which is in line with the resource needs of the Good Life Framework [7]. 

It is for the reasons described above that the authors believe the Good Life Framework to be the best framework for practical on-farm assessment of positive welfare and have opted to extend the Good Life Framework to other farmed species.

At present, even higher welfare farm assurance such as those in the Global Animal Welfare Assurance alliance (https://gawassurance.org/, accessed on 23 January 2022) have a limited focus on positive welfare in their standards. Therefore, the projected use of the Good Life Framework is within farm assurance scheme standards to enable the assurance of positive welfare and a good life for animals on certified farms. 

The aim of this work is to describe the initial development of Good Life Frameworks for beef cattle (*Bos taurus*), pigs (*Sus scrofa* domesticus) and broiler chickens (*Gallus gallus domesticus*). These three species were chosen because they are three of the main farmed species worldwide, and the development of Good Life Frameworks for dairy cattle and sheep has already been commenced by researchers at the Royal Agricultural College and Scotland’s Rural College (UK), although these currently remain unpublished. This initial development stage included the piloting of the frameworks on a small number of farms, working with the UK farm-assurance industry in order to ensure that the frameworks were practical for on-farm assessment. The aim of the piloting phase was not to assign scores to each farm-assurance scheme based on the level of positive welfare opportunities provided by the sample of producers, but rather to test the frameworks on-farm and refine accordingly.

## 2. Materials and Methods

### 2.1. Literature Review

The first stage in developing Good Life Frameworks for beef cattle, broiler chickens and pigs was to carry out a literature review for each species in each of the 12 resource needs categorised under the five Good Life Opportunities of comfort, pleasure, confidence, interest and healthy life, as outlined by Edgar et al. [7] (see Table 1).

In the original generic Good Life Framework blueprint, there was a 13th resource need in the good life opportunity of comfort: A safe environment, which for the laying hen framework pertained to the safety of perches. The current authors chose to remove this resource need as it does not pertain to positive welfare but rather the protection from harm (preventing negative welfare). For beef cattle, the generic resource need “enhanced learning opportunities” (which covers increased complexity of environment) was changed to “pasture choices.” For broiler chickens, we added to the generic Good Life Framework blueprint the resource need of “promoting ranging” in the good-life opportunity of interest (which was also included in the laying hen Good Life Framework), and under the good-life opportunity of a healthy life, we added the resource needs of “dustbathing opportunities” and “resting opportunities,” representing important and motivated behaviours [18,19] for broilers in commercial systems, which were not included in the generic blueprint of a Good Life Framework.

We used Scopus as our literature database. For each species, we carried out searches for positive welfare (e.g., search terms: Pig AND positive welfare), for each good-life opportunity (e.g., pig AND comfort; pig AND confidence; pig AND pleasure; pig AND interest), and each resource need (e.g., pig AND physical comfort; pig AND thermal comfort; pig AND food enrichment etc). We also searched for resources that fulfilled each resource need (e.g., pig AND bedding; pig AND toys) and resources that promoted positive behaviours pertaining to each good-life opportunity (e.g., pig AND play; pig AND rooting). 

From the resulting literature, we examined each paper for:

(1) Resources that fulfilled each resource need;
(2) Resources that promoted positive behaviours pertaining to each good life opportunity;
(3) Resources that animals showed a preference for.

For example, for pigs, for the resource need ‘enriched environment’ within the Good Life Opportunity of Interest, the literature was searched for:

(1) Environmental enrichment for pigs (fulfilling the resource criterion of an enriched environment).
(2) Resources that promoted the interest-related positive behaviour of exploration and foraging using environmental enrichment.
(3) Resources that pigs preferred to use and forage in.

Resources in this context included both materials (for example, bedding, toys, feed) and management practices (e.g., providing outdoor access, stockkeeper training, handling practices). 

### 2.2. Framework Development

Based on these literature reviews and the blueprint of a Good Life Framework provided by Edgar et al. [7], we developed a framework consisting of three tiers of increasingly positive welfare: Welfare +, Welfare ++, Welfare +++. Each tier contained resources that aimed to incrementally increase the opportunities for a good life over and above the requirements of UK law and code of practice. The process of assigning resources to each of the three tiers began with establishing the highest tier (Welfare +++) as the ideal environment and management for the species in question, then working backwards to Welfare + as a modest increase in positive welfare opportunities beyond UK law and code of practice requirements. The increase in positive welfare from tier to tier involved providing animals with:

(1) More resources (and therefore more opportunities to experience positive welfare).
(2) Resources of increasing preference.
(3) Increasing choice in resources.

For broiler chickens, resources for laying hens from Edgar et al. [7] were used as a foundation for tier development, as laying hens and meat chickens are the same species, and therefore can be considered to have the same species-specific needs. However, both layers and broilers have undergone intense selective breeding in the past few decades, leading to divergence in their phenotypes, so resource recommendations were adapted to take this divergence into account using the available scientific literature on broilers.

Likewise, for pigs, the ‘pig-keeping scores’ of Mullan et al. [20] were used as a foundation and further developed by the literature review.

Finally, for beef cattle, currently unpublished good life resource tiers for dairy cows that have undergone expert consultation were used as a foundation for the resource tiers, as beef and dairy cattle are the same species and therefore have the same species-specific needs. Although the dairy cow good-life framework is currently unpublished, all references used to construct the framework are published and included in this paper.

The scoring system of the framework follows that set out by Edgar et al. [7]; for each resource need, farms are scored between 0 and 3 based on whether they provide all the resources in each positive welfare tier (0 = did not provide all resources in Welfare +, 1 = provided all resources in Welfare +, 2 = provided all resources in Welfare ++, 3 = provided all resources in Welfare +++). Scores are incremental, i.e., producers cannot achieve Welfare ++ (score 2) if they did not meet all the requirements of Welfare +; they could not achieve Welfare ++ (score 3) if they did not meet all the requirements of Welfare + and Welfare ++.

### 2.3. Stakeholder Feedback

Stakeholders who had extensive experience and knowledge of pig, cattle and chicken welfare, as judged by their professional position, qualifications, reputation and experience of commercial farming were sent the Good Life Frameworks to review and give their input. These were stakeholders from the Pasture-Fed Livestock Association (UK), RSPCA (UK), Dierenbescherming (Netherlands) and the Soil Association (UK). These organisations were selected as a representation of the farm-assurance industry and those which had the time to review the frameworks within the timeframe of the study. The frameworks underwent revisions based on the stakeholders’ feedback.

### 2.4. Piloting

The frameworks were piloted between March and September 2021 on a sample of farms across the UK for each species via the three main farm-assurance schemes in the UK, referred to here as Farm Assurance Scheme A, Farm Assurance Scheme B and Farm Assurance Scheme C.

The broiler framework was piloted on Farm Assurance Scheme A member farms, the pig framework on Farm Assurance Scheme B farms and the beef cattle frameworks on Farm Assurance Scheme C farms. Each farm-assurance scheme sought a sample of around 10 farms to pilot the framework. This was deemed a reasonable sample size based on the scope of the project, the timeframe available and the existing workload of the assessors and member farmers. The frameworks were completed either as part of an on-farm audit carried out by an assessor or by the producer as a self-assessment. There was only one assessor present per audit, and not always the same assessor each time. For the purpose of this pilot, no training was given to assessors or producers on using the framework. The farm-assurance schemes received consent from the producers for their participation in the project. 

For each resource need, farms were scored between 0 and 3 (see Framework Development for an explanation of the scoring system).

Based on the scores achieved by the producers, and any feedback from the producers and/or assessors, refinements were made to the Good Life Frameworks, which are detailed in the Results and Discussion sections.

### 2.5. Ethical Approval

This project falls into the classification of Patient and Participant Involvement (PPI), whereby engagement from key stakeholders (producers and farm-assurance scheme assessors) was undertaken to evaluate the Good Life Framework to inform future research. A formal research ethics review was not required. Nonetheless, good research principles were adhered to by providing producers with a participant information sheet detailing the background and purpose of the piloting, explaining that participation is entirely voluntary and that all data will be anonymised so that there is no information that can be used to identify participating farms or individuals. 

## 3. Results

### 3.1. Framework Development

The frameworks resulting from the literature review and stakeholder feedback, which were used in the piloting, can be seen in Supplementary Materials Tables S1–S3.

### 3.2. Piloting

#### 3.2.1. Broilers—Farm Assurance Scheme A

A total of 10 producers participated in the piloting. Table 2 describes the farming systems in this sample and whether the Good Life Framework was completed by an assessor or the producer. 

Table 3 shows the number of producers that scored either 0, 1, 2 or 3, and the median score for the sample for each resource need. Because none of the participating farms were breeding units, the breeding and nurturing experiences were not applicable (“N/A” in the table).

#### 3.2.2. Pigs—Farm Assurance Scheme B

A total of 12 producers participated in the piloting exercise. Table 4 describes the farming systems in this sample and whether the Good Life Framework was completed by an assessor or the producer. 

Table 5 shows the number of producers that scored either 0, 1, 2 or 3, and the median score for the sample for each resource need.

#### 3.2.3. Beef Cattle—Farm Assurance Scheme C

A total of 10 producers participated in the piloting. Table 6 describes the farming systems in this sample and whether the Good Life Framework was completed by an assessor or the producer. 

Table 7 shows the number of producers that scored either 0, 1, 2 or 3, and the median score for the sample for each resource need.

### 3.3. Feedback

Table 8 demonstrates the feedback received from producers and assessors for each Good Life Framework.

### 3.4. Framework Refinement

Following the piloting exercise, the main refinements were to re-word questions that proved unclear and difficult to answer. For example, some questions contained double negatives, which proved confusing.

Based on the results, we re-examined any resources that none of the participating producers were providing. If we felt that the scientific evidence from the literature review was sufficiently strong enough to defend those resources, we left them in; if not, we removed them. This was mainly for resource provision based on offering choice of multiple resources.

The resource of “increased space” in the broiler framework was highlighted as subjective to judge; we therefore specified a maximum stocking density of 30 kg/m^2^ [21].

Finally, we streamlined resource needs that contained many resources by combining related resources as alternative examples. 

The refined frameworks can be seen in Supplementary Materials Tables S4–S6.

## 4. Discussion

Results of the initial piloting of Good Life Frameworks to assess positive welfare opportunities for pigs, beef cattle and broiler chickens have revealed support from the farm assurance industry in the usefulness of such an on-farm tool and have allowed for useful refinement of the frameworks. The frameworks require further validation if they are to be used for farm accreditation purposes, which is what we hope this work will stimulate. 

The Good Life Framework scores are based on scientific evidence of the resources required to provide each species with increasing opportunities for positive welfare. The aim of this study, however, was not to assign scores to each farm assurance scheme based on the level of positive welfare opportunities provided by the sample of producers, but rather to test the frameworks on-farm and refine accordingly. Nonetheless, results of the piloting exercise demonstrate that some producers are going above and beyond legislation and code to provide positive welfare for their animals. In support of this, in an analysis of 49 non-caged laying hen flocks using the laying hen Good Life Framework, 63% of flocks were given a score of 1 (Welfare +) or above [22]. This provision of positive welfare opportunities currently has no reward mechanism, including direct financial benefit, in the current market.

Piloting the broiler Good Life Framework with Farm Assurance Scheme A demonstrated that, on average (median scores), the sample of broiler producers belonging to farm assurance scheme A provided opportunities for positive welfare at the UK minimum legislation and welfare code, and exceeded in the resource needs of a comfortable thermal environment (median score 1), positive social interactions (median score 1), dustbathing opportunities (median score 1) and resting opportunities (median score 2). This was to be expected as most of the participating farms were intensive indoor systems, which sit at the baseline of permissible animal welfare as dictated by UK law and provide little in the way of varied environmental choices. However, between one and three farms in the sample (the higher-welfare indoors, free-range and organic free-range systems) achieved scores of 2 and 3 for some resource needs (comfortable thermal environment, play opportunities, positive social interactions, enhanced learning opportunities, dustbathing opportunities, management for positive health and positive genetic selection). 

For the sample of pig producers belonging to Farm Assurance Scheme B, almost half of the resource needs had a median score above 0: Comfortable physical environment (median score 1), comfortable thermal environment (median score 1), positive experiences with stockpersons (median score 2), management policy for positive health (median score 3) and positive genetic selection for long-term health and welfare (median score 1.5). There were between one and seven producers scoring 2 or 3 for some of the resource needs (comfortable physical and thermal environment, positive experiences with stockpersons, positive social interactions, enriched environment, management for positive health, positive genetic selection for long-term health and welfare and promoting a natural body type).

Of the sample of beef cattle producers belonging to Farm Assurance Scheme C, the only resource tier that had an average (median) score of 0 was promoting a natural body type. All the other resource needs had an average median score of between 1.5 and 2.5, suggesting that this sample of beef producers were going well above and beyond UK minimum legislation and welfare codes in providing opportunities for positive welfare.

It should be noted that the majority of beef in the UK is produced in outdoor extensive systems, which entails greater environmental choice, making it likely that beef farms belonging to any of the three assurance schemes would have scored highly. However, the fact all 10 beef cattle farms were organic systems, and organic standards have higher standards of animal welfare, may explain the high scores achieved by this sample of producers.

It should also be noted that inter- and intra-observer reliability when scoring was not tested in the piloting exercise. An independent auditor may score a farm differently than the producers, an experienced auditor may score differently to an inexperienced auditor and it is possible that self-assessment scores may be biased towards higher scores. This presents a limitation of the study; however as stated, the aim was not to produce reliable scores. For future use of the framework in assessing positive welfare, inter- and intra-observer reliability should be measured, including comparisons between auditors and producers, and those carrying out the assessment should receive training in using the framework.

Direct feedback on the frameworks was not received from all 32 participating producers or all assessors. A lack of feedback could suggest that the producers did not feel strongly, either positively or negatively, about the frameworks. Nonetheless, the feedback received suggested that both producers and assessors were generally positive about the frameworks and their usefulness in assessing and assuring positive welfare on farms. 

One broiler producer felt that the broiler Good Life Framework was mostly applicable to layers and free-range systems and therefore did not capture what they were doing in their higher-welfare indoor system. However, according to our literature review on resources needed to provide increased opportunities for positive welfare through comfort, confidence, interest and pleasure for broiler chickens, outdoor access (i.e., free-range systems), perching opportunities and other enrichments (usually only included in laying hen standards, not broilers) need to be included in the Good Life Framework. This may have led to the participant’s perception that the framework seemed less applicable to their indoor-only system. 

Nonetheless, Good Life Frameworks for all species were developed to be applicable to both intensive and extensive systems; however, extensive systems, providing more freedom, choice and enrichment for animals, are able to achieve higher scores than intensive systems.

The feedback from the auditors of Farm Assurance Scheme B, which reflected feedback from the pig producers they spoke to, was very positive, apart from the wording of some elements of the framework, which was taken into account, plus the achievability of some resources in a commercial setting, also taken into account (see below).

One auditor of Farm Assurance Scheme C supported the idea that the Good Life Framework could be incorporated into a normal auditor inspection but suggested that only Welfare +++ need be included, as most producers were meeting Welfare + and Welfare ++. The auditor questioned whether some resources in Welfare +++ could ever be provided in a commercial setting.

Both the feedback and piloting results led to the useful refinement of all three frameworks. The aim in assigning resources to each of the three tiers (Welfare +, Welfare ++, Welfare +++) was that the highest tier should represent the ideal environment and management of farm animals, regardless of barriers to achieving this level in current commercial systems. However, for Welfare + and Welfare ++, the aim was to balance achievability in a higher welfare commercial setting with scientific evidence. The main refinements were made to the provision of environmental choice. For example, for the resource need of a comfortable physical environment for pigs, a requirement of Welfare ++ was that at least two different types of bedding were provided. No producers in the sample, despite being members of a welfare-focussed assurance scheme, achieved this criterion. Choice of resources was an important element in the frameworks, but the choice of bedding was not deemed sufficiently vital to prevent producers from achieving good positive welfare scores if they were providing animals with resources to allow for good physical comfort. Often, this element of choice was moved to the top tier (Welfare +++) or removed entirely until future preference tests could show that the species in question showed individual preferences for the resource in question.

The piloting exercise also led to the realisation that producers were hindered in achieving good welfare scores when a resource need contained a long list of different resources that were in fact all related to the same concept. For example, in the pig framework, the resource need of an enriched environment originally included a long list of different types of enrichment resources identified from the literature, in each tier. This was condensed around the core concepts of providing a rootable substrate (Welfare +), providing object enrichment (Welfare ++) and providing objects of improved quality and retaining novelty of objects (Welfare +++), with the different types of resources listed as alternatives (using the wording ‘or’) rather than separate requirements.

For farmers that were not already providing the resources needed for positive welfare opportunities according to the Good Life Framework, strategies could be implemented to encourage such resource provision, such as government payment incentives, improved marketing opportunities and the Good Life Framework being included in farm assurance scheme standards, in part or in its entirety, so that resource provision would be required to achieve certification.

All participating farm assurance schemes expressed interest in the continued development and potential future uses of these Good Life Frameworks as a way of assuring positive welfare provided by their members.

Piloting the newly refined frameworks could provide a more accurate picture of the positive welfare opportunities on UK farm-assured farms, and the next step in this work would be to pilot the refined frameworks on a larger sample of farms. The frameworks in their existing form also provide a useful tool to engage with producers in discussions on positive welfare. However, if the frameworks are to be used for accreditation purposes, then validation of whether the resources included within the framework do result in positive welfare for the animals in question is needed. Part of the development process of the Good Life Frameworks was to identify scientific evidence for resources that have been shown to elicit positive behaviours, and therefore a certain level of validation could be considered to already exist. However, the frameworks should be validated in their entirety, not only in terms of whether each resource in the framework does increase positive welfare but also whether the resources in tier Welfare +++ increased positive welfare more than those in Welfare ++ and Welfare + (thus validating the assignment of resources to each tier). This could be achieved, for example, by assessing whether resource provision elicits positive behaviours pertaining to each of these good-life opportunities (for example, play and exploration), and the use of other behavioural indicators of positive welfare, for example, in broiler chickens such behaviours include worm-running (running with an object held in the beak), play-fighting, wing-flapping, jumping, running, ground-scratching, vertical wing shaking and perching [21]. Some animal-based measures show promise as positive welfare indicators, which could be used to validate the framework, such as behavioural diversity [23] and vocalisations [24]. However, as previously discussed, both these and other proposed measures require further research and validation [23,24]. Recently, an investigation in farmed rainbow trout (*Oncorhynchus mykiss*) used a nature-, functions- and feelings-based approach to validate the positive welfare effects of environmental enrichment for the fish, where nature refers to the promotion of natural behaviours, function refers to the maintenance of biological functions and feelings being the stimulation of exploration and curiosity [25]. Validation of the resource provisions stipulated in the Good Life Framework should be the focus of future work. 

## 5. Conclusions

This work describes the initial stage of development of “Good Life Frameworks” for beef cattle, pigs and broiler chickens as a way of assessing opportunities for positive welfare in these species. Partnering with the UK farm-assurance industry, which represents a large proportion of the UK farming industry, has ensured that this development stage is relevant to the real-life applicability of these frameworks as on-farm tools to assess positive-welfare opportunities for farm animals. This paper brings the frameworks into the public domain in order to stimulate and facilitate further development of and research using these frameworks.

## Figures and Tables

**Table 1:** Twelve resource needs categorised under the five Good Life Opportunities.

Good Life Opportunity	Resource Need
Comfort	Comfortable physical environment
	Comfortable thermal environment
	Safe environment
Pleasure	Food enrichment
	Play opportunities
	Breeding and nurturing opportunities
Confidence	Positive experiences with people
	Positive social interactions
Interest	Enriched environment
	Enhanced learning opportunities
Healthy life	Management policy for positive health
	Breeding for positive welfare
	Promoting a natural body type (telos)

**Table 2:** Farming system of a sample of 10 broiler farms and the assessment type carried out.

Farming System	Number of Farms in Sample
Indoor intensive ^a^	7
Indoor higher welfare ^b^	1
Free range	1
Organic free range	1
Assessment type	Number of farms in sample
Audit by assessor	4
Self-assessment by producer	6

^a^ Indoor intensive systems are indoor sheds certified to meet, and in places exceed, minimum GB legislation in terms of stocking density and lux, with additional standards on the provision of environmental enrichment. ^b^ Indoor higher welfare systems have lower stocking density, increased provision of environmental enrichment, natural light and the use of slower-growing breeds.

**Table 3:** Number of broiler producers from a sample of 10 belonging to Farm Assurance Scheme A that scored either 0, 1, 2 or 3, and the median score for the sample for each resource need.

Resource Need	0	1	2	3	Median
Comfortable physical environment	10	0	0	0	0
Comfortable thermal environment	4	4	1	1	1
Food enrichment	10	0	0	0	0
Play opportunities	7	0	2	1	0
Breeding and nurturing experiences	N/A	N/A	N/A	N/A	N/A
Positive experience with stock keepers	10	0	0	0	0
Positive social interactions	1	8	1	0	1
Positively enriched environment	10	0	0	0	0
Enhanced learning opportunities	8	0	2	0	0
Promoting ranging	8	2	0	0	0
Dustbathing opportunities	4	5	1	0	1
Resting opportunities	4	0	6	0	2
Management policy for positive health	7	0	1	2	0
Positive genetic selection for long term health and welfare	6	1	0	3	0

**Table 4:** Farming system of a sample of 12 pig farms and the assessment type carried out.

Farming System	Number of Farms in Sample
Indoor finishing	8
Indoor rearing	2
Outdoor breeding	1
Outdoor breeding with indoor finishing	1
Assessment type	Number of farms in sample
Audit by assessor	9
Self-assessment by producer	3

**Table 5:** Number of pig producers from a sample of 12 belonging to Farm Assurance Scheme B that scored either 0, 1, 2 or 3, and the median score for the sample for each resource need.

Resource Need	0	1	2	3	Median
Comfortable physical environment	3	6	0	3	1
Comfortable thermal environment	4	7	0	1	1
Food enrichment	11	1	0	0	0
Play opportunities	12	0	0	0	0
Breeding and nurturing experiences	2	1	0	0	0
Positive experience with stockpersons	1	4	7	0	2
Positive social interactions	8	2	2	0	0
Enriched environment	10	1	1	0	0
Enhanced learning opportunities	12	0	0	0	0
Management policy for positive health	0	0	5	7	3
Positive genetic selection for long-term health and welfare	3	1	3	1	1.5
Promoting a natural body type (telos)	8	2	0	2	0

**Table 6:** Farming system of a sample of 10 beef cattle farms and the assessment type carried out.

Farming System	Number of Farms in Sample
Organic	10
Assessment type	Number of farms in sample
Audit by assessor	1
Self-assessment by producer	9

**Table 7:** Number of beef cattle producers from a sample of 10 belonging to Farm Assurance Scheme C that scored either 0, 1, 2 or 3, and the median score for the sample for each resource need.

Resource Need	0	1	2	3	Median
Comfortable physical environment	1	3	2	4	2
Comfortable thermal environment	1	1	7	1	2
Play opportunities	0	2	3	5	2.5
Breeding and nurturing experiences	0	5	1	4	1.5
Food enrichment	1	3	2	3	2
Positive experience with stock keepers	3	0	6	1	2
Positive social experiences within the herd	2	0	3	5	2.5
Enriched environment	4	0	3	3	2
Pasture choices	0	0	5	5	2.5
Management policy for positive health	1	4	3	2	1.5
Positive genetic selection for long term health and welfare	0	1	6	3	2
Promoting a natural body type (telos)	8	2	0	0	0

**Table 8:** Feedback received from producers and assessors for each Good Life Framework.

Feedback	Broiler Good Life Framework	Pig Good Life Framework	Beef Cattle Good Life Framework
Producer feedback	“I did not feel the questions really explored what we are doing on the farm. The farm grows chicken to RT Enhanced Welfare Standards so levels of perches etc are controlled by the standard. Feel form needs to be specific for farm types. A great deal of form was applicable to layers and Free Range.”	Some producer feedback via assessors (see below)	None
Auditor Feedback	None	“Group reps and farmers I have found all of them to be positive and willing to contribute to something which we can all learn from and ultimately improve welfare. The more negative feedback was around the wording which could be clearer on some points.”	“It struck me organic farms would all meet the baseline and second level of test, while the above areas it would vary from farm to farm, and it would need to be considered some of the questions would never be met, or need more clarification. I am sure the assessment could be incorporated into a normal organic inspection. However I think it should be amended to just test the Welfare+++ sections only, saving time, as majority of organic units would meet lower levels by default.”
		“On the whole, I thought some of the questions in the framework were very good, and would lead to a high level of welfare if they were implemented on farms. However, I felt that a few of the questions were poorly worded or repetitive.”	
		“In general farmers were happy and always interested in what people are looking at. Some farmers felt some of the measures where not realistic in what could be achievable in a commercial set up such as the piglets being with sows for 50 days. I really like the idea of measures, the wording was hard to understand with the double negative so having to read it 2 or 3 times to work out!”	

## Data Availability

Data is contained within the article. Further data are not publicly available due to confidentiality: participants were assured that data would be anonymised so that no information can be used to identify participating farms or individuals.

## Supplementary Materials

The following supporting information can be downloaded at: https://www.mdpi.com/article/10.3390/ani12050565/s1, Table S1: Beef cattle Good Life Framework resulting from the literature review and expert stakeholder feedback, which was used in the piloting exercise; Table S2: Broiler Good Life Framework resulting from the literature review and expert stakeholder feedback, which was used in the piloting exercise, Table S3: Pig Good Life Framework resulting from the literature review and expert stakeholder feedback, which was used in the piloting exercise, Table S4: Beef cattle Good Life Framework resulting from refinement after the piloting exercise, Table S5: Broiler Good Life Framework resulting from refinement after the piloting exercise, Table S6: Pig Good Life Framework resulting from refinement after the piloting exercise.
