# Supplementary material for: Advancing a “Good Life” for Farm Animals: Development of Resource Tier Frameworks for On-Farm Assessment of Positive Welfare for Beef Cattle, Broiler Chicken and Pigs
Source: Animals (Basel). 2022 Feb 23;12(5):565. doi: 10.3390/ani12050565 (PMC8908822; doi:10.3390/ani12050565)
Supplement: Supplementary file 1 [file animals-12-00565-s001.zip › S1 Beef cattle Good Life Framework pre pilot.pdf]

Table S1. Beef cattle Good Life Framework resulting from the literature review and expert stakeholder feedback which was used in the piloting exercise.

Good Life framework – Please answer each of the questions with Y (yes) or N (no) or N/A if not applicable

<table>
<tr><th colspan="6">Good Life Opportunity Comfort By choice of physical environment</th></tr>
<tr><th colspan="6">Objective Cattle should be able to exercise individual preferences for their physical comfort at all times.</th></tr>
<tr><th>Welfare +</th><th>Y/N:</th><th>Welfare + +</th><th>Y/N:</th><th>Welfare + + +</th><th>Y/N:</th></tr>
<tr><td>+ Cattle are not tethered on the farm - is this true? [1,2]</td><td></td><td>++ Are cattle loose housed with free access to an unrestricted/undefined lying area that allows the cattle the choice to lie in any orientation and to switch sides easily?</td><td></td><td>+++ Is there at least 10% -20% more lying space than cattle, to allow cattle choice of where to lie and who to lie next to?</td><td></td></tr>
<tr><td rowspan="2">+ If cattle are housed some or all of the time in sheds with slatted floors, are the slats covered with rubber matting? [3,4]</td><td rowspan="2"></td><td>++ There are no sheds on the farm with slatted flooring – is this true? [5]</td><td></td><td rowspan="2">+++ Is cattle comfort monitored (using any of the following: cameras, activity meters, behaviour observations, welfare outcome scores) and resources/ management practices altered to improve comfort where necessary?</td><td rowspan="2"></td></tr>
<tr><td>++ Is bedding material e.g. straw kept deep (compressible), clean and dry i.e. by regular renewal? [3,4,6,7]</td><td></td></tr>
<tr><td colspan="6">Any comments or feedback on any of these questions:</td></tr>
</table>

<table>
<tr><th colspan="6">Good Life Opportunity Comfort By choice of thermal environment</th></tr>
<tr><th colspan="6">Objective Cattle should be able to exercise individual preferences for their thermal comfort at all times.</th></tr>
<tr><th>Welfare +</th><th>Y/N:</th><th>Welfare + +</th><th>Y/N:</th><th>Welfare + + +</th><th>Y/N:</th></tr>
<tr><td>+ Do housed cattle of all ages have a choice of temperatures (nearer to or further from open or ventilated areas) and can they always avoid thermal extremes (e.g. fans and ventilation for heat, no excessive drafts in cold weather)? [8]</td><td></td><td>++ Can housed cattle choose to be indoors or outdoors for at least part of the day/night? [9]</td><td></td><td>+++ When outdoors/grazing is there a choice of temperatures/shade intensities (more shade/less shade) i.e. high hedges, trees and /or artificial structures? [10,11]</td><td></td></tr>
<tr><td rowspan="3">+ Is there sufficient dry bedding, renewed regularly, to insulate cattle during cold? [8]</td><td rowspan="3"></td><td>++ Is there shelter available for at least some animals e.g. hedges, trees, sheds, walls, portable structures? [8]</td><td></td><td rowspan="3">+++ Is the potential for heat stress monitored using technology and actions taken to reduce heat stress?</td><td rowspan="3"></td></tr>
<tr><td>++ Is sufficient shelter provided for all animals to stand or lie in the shelter and shade at the same time?</td><td></td></tr>
<tr><td>++ Is there provision of sufficient outdoor water sources during hot periods? [8]</td><td></td></tr>
<tr><td colspan="6">Any comments or feedback on this opportunity:</td></tr>
</table>

Good Life Opportunity **Pleasure** by play					
**Objective Cattle should be able to excercise individual preferences for play.**					
**Welfare +**	**Y/N:**	**Welfare ++**	**Y/N:**	**Welfare +++**	**Y/N:**
NO QUESTION FOR THIS TIER		++ Do cattle of all ages have enough space, appropriate design of housing/pasture (layout and floor surface) to allow **some** opportunities for play including running/jumping/chasing and social play behaviour?		+++ Do cattle of all ages have the opportunity to move at different paces and for social play available **at all times?** (excluding calving, hospitalisation due to disease)	
**Any comments or feedback on this opportunity:**					

Good Life opportunity pleasure By breeding and nurturing experiences

Objective Cattle should be able to have positive reproductive and nurturing experiences.

**Welfare +**	**Y/N:**	**Welfare ++**	**Y/N:**	**Welfare +++**	**Y/N:**
+ Are calves kept with their dam until at least 45 days old? [12]		++ Are calves kept with their dam for at least 3 months old? [12]		+++ Are calves kept with their own dam until natural weaning (7 to 10 months)? [5]	
+ Are calves separated during weaning using fence line separation? [12]		++ Is gradual weaning carried out i.e. two-step weaning – suckling prevention (anti- suckling devices, restricted suckling periods then fence line separation to allow physical and social contact? [12]			
+ Is solid food offered to calves from a few days of age? e.g. concentrates, grazing/ forage. [12]		++ During periods of separation, are calves are kept in a familiar environment with familiar individuals OR grouped according to body size? [12]			
+ Are calves kept in stable groups of familiar individuals after separation from the dam? (NB they could still be fed milk as in dairy beef enterprises). [12]					

Any comments or feedback on this opportunity:

<table>
<tr><th colspan="6">Good Life Opportunity Pleasure by food enrichment</th></tr>
<tr><th colspan="6">Objective Cattle should be able to exercise individual preferences for type of food and how it is obtained.</th></tr>
<tr><th>Welfare +</th><th>Y/N:</th><th>Welfare ++</th><th>Y/N:</th><th>Welfare +++</th><th>Y/N:</th></tr>
<tr><td>+ Are cattle offered, from weaning, sufficient amount of nutritionally balanced diet consisting of fibrous/ forage rather than high concentrate feed? [13,5]</td><td></td><td rowspan="2">++ Are cattle provided with some variety in their diet indoors? i.e. at least two types of forage (grass silage, maize silage, hay, wholecrop silage, straw, turnips or fodder beet), bales and clamp silage, coarsely mixed TMR, long fibre and/or food presented in different/novel places/ways to facilitate exploration and seeking behaviour e.g. choice of feed fences/areas, ring feeders, troughs. [15]</td><td rowspan="2"></td><td>+++ Are cattle offered a choice of pasture herbage outdoors e.g. clover, diverse leys, more species than just ryegrass in the sward.</td><td></td></tr>
<tr><td>+ When housed, are cattle provided with long fibre, such as straw or hay, to allow exploratory behaviour with their mouths e.g. manipulation, licking, chewing, ruminating? [14]</td><td></td><td>+++ Are cattle provided with a choice of forage/ concentrate rations in the field, or are these presented in different/novel places/ways? e.g. use of ring feeders/ feed trailers/ troughs on legs/on ground.</td><td></td></tr>
<tr><td colspan="6">Any comments or feedback on this opportunity:</td></tr>
</table>

<table>
<tr><th colspan="6">Good Life Opportunity <u>Confidence</u> <u>By positive experience with stock keepers</u></th></tr>
<tr><th colspan="6"><u>Objective</u> Cattle should be able to have positive experience of people when encountered.</th></tr>
<tr><th>Welfare +</th><th>Y/N:</th><th>Welfare ++</th><th>Y/N:</th><th>Welfare +++</th><th>Y/N:</th></tr>
<tr><td>+ Goads are never used on the farm when handling cattle - is this true?</td><td></td><td>++ Are young cattle trained using positive reinforcement (positive handling, attention, food) from a young age to move around calmly (when led, driven or being examined)? [16]</td><td></td><td>+++ Do stock keepers undergo formal training e.g. on practical welfare techniques, understanding cattle behaviour and good handling practices? [16,17]</td><td></td></tr>
<tr><td rowspan="2">+ Does the design and layout of handling systems encourage natural, safe movement of animals (e.g. suitable race with wider funnel at the start, ‘sweeping’ gate behind, curves in the race, boarded sides) and/or are animals very familiar with the handling system that is available and exhibit low stress when handled? [5]</td><td rowspan="2"></td><td>++ Do stock persons spend time talking to, scratching heads/tails of cattle?</td><td></td><td rowspan="3">+++ Do stock keepers undergo formal training to develop positive attitudes towards cattle e.g. through cognitive behavioural training? [18]</td><td rowspan="3"></td></tr>
<tr><td>++ Is this extra time spent on positive interactions maintained throughout life of the cattle i.e. from calves to older cattle? [16]</td><td></td></tr>
<tr><td>+ Do stock persons spend time regularly in the presence of cattle of all ages? [16]</td><td></td><td>++ Are calm adult cattle present in groups of young cattle?</td><td></td></tr>
<tr><td colspan="6">Any comments or feedback on this opportunity:</td></tr>
</table>

Good Life Opportunity Confidence By positive learning, resilience and social experiences within the herd					
**Objective** **Cows confidence should be promoted by the opportunity to have positive social experiences within the herd, including establishment and maintenance of a stable social hierarchy and being able to learn about their environment from other cattle.**					
**Welfare +**	**Y/N:**	**Welfare ++**	**Y/N:**	**Welfare +++**	**Y/N:**
+ Are cattle kept in such a way that they can maintain small, stable social groups of familiar individuals, no more than 70 animals to enable individual recognition? [19]		++ Are calves/youngstock kept and reared together from the time they are separated from their dams or arrive at the farm?		+++ Does a bull run with the herd, some or all the time (including sweeper bull / vasectomised bull) allowing natural behaviour to be expressed and natural social hierarchy and herd stability to be maintained?	
+ Are calves kept in familiar groups OR grouped according to size? [12]				+++ Are breeding bulls isolated only for biosecurity/ disease screening and at all other times kept with or in sight of other stock, providing safety can be maintained? [5]	
**Any comments or feedback on this opportunity:**				+++ Do feeding troughs/feed bunkers have divisions [20]	
				+++ Is there enough space at the feed fence to ensure there is not competition for feed/ bullying? [19]	

**Good Life Opportunity Interest By a positively enriched environment**					
**Objective Cattle should have opportunities to explore and play in an enriched environment throughout their lives.**					
**Welfare +**<br>Some enrichment	**Y/N:**	**Welfare ++**<br>Continuous enrichment	**Y/N:**	**Welfare +++**<br>Continuous varied enrichment	**Y/N:**
+ is there a choice between 2 or more positive activities for exploration provided occasionally?<br>(E.g. hay balls, cardboard boxes, automatic or manual brushes [21], padded posts, sand pit, hay bales, tree stumps, hedges, tractor tyres)?		++ Are there opportunities for choice between two (or more) positively stimulating activities (see examples in welfare +) provided continuously throughout life?		+++ Are there opportunities for choice between two (or more) positively stimulating activities, provided continuously and changed or replenished frequently throughout life? (i.e. between every 2 weeks and 3 months).	
**Any comments or feedback on this opportunity:**					

Good Life Opportunity **Interest** By pasture choices					
**Objective** **Interest should be promoted by the opportunity to be able to exercise individual preferences about their access to and use of pasture.**					
**Welfare +**<br>Some pasture access	**Y/N:**	**Welfare ++**<br>Pasture access	**Y/N:**	**Welfare +++**<br>Extended pasture access	**Y/N:**
+ Do cattle have access to pasture during **parts of the day/night** during the summer grazing season [22,23]		++ Do all cattle have choice to access pasture at **all** times of day/ night during the summer grazing season?		+++ Do cattle have easy access to sheltered pasture (trees, hedges, artificial shelter) at all times of day/ night across an **extended** grazing season i.e. at least 180-200 days a year?	
+ in low rainfall locations i.e. less than 28 inches annual rainfall, free draining soil types, do all cattle have access to straw corrals with a pad of well drained bedding (e.g. woodchip) during parts of the day and night? [26]				+++ Are both access and shelter sufficient to allow pasture to be safely used in winter, with efforts to minimise mud patches/ poaching of high traffic areas? [5]	
**Any comments or feedback on this opportunity:**					

<table>
<tr><th colspan="6">Good Life Opportunity <u>Healthy Life</u> <u>By management policy for positive health</u></th></tr>
<tr><th colspan="6"><u>Objective</u> Stock persons should manage day to day cattle health effectively.</th></tr>
<tr><th>Welfare +</th><th>Y/N:</th><th>Welfare ++</th><th>Y/N:</th><th>Welfare +++</th><th>Y/N:</th></tr>
<tr><td>+ Is a health and welfare plan implemented and reviewed frequently? i.e. every 6 months or more often and corrective action taken?</td><td></td><td>++ Do stock keepers act on changes in behaviour within 24 hours?</td><td></td><td rowspan="3">+++ Do farm staff take part in welfare improvement activities with wider benefits? i.e. member of welfare policy/advisory group, peer advisor, welfare discussion groups, participates in on-farm welfare research/innovation.</td><td rowspan="3"></td></tr>
<tr><td>+ Are medicines used as required, in conjunction with good management, rather than routinely or blanket approach basis?</td><td></td><td>++Do stock keepers know the individual habits and preferences of every animal in the herd?</td><td></td></tr>
<tr><td>+ Do stock keepers recognise and investigate changes in cattle behaviour for health and welfare reasons within 24 hours?</td><td></td><td>++ Is there regular dialogue with the vet (and/or scheme welfare advisor if applicable)?</td><td></td></tr>
<tr><td colspan="6">Any comments or feedback on this opportunity:</td></tr>
</table>

<table>
<tr><th colspan="6">Good Life opportunity Healthy life By positive genetic selection for long term health and welfare</th></tr>
<tr><th colspan="6"><u>Objective</u> Stock keepers should positively influence the long term health and welfare of cattle</th></tr>
<tr><th>Welfare +</th><th>Y/N:</th><th>Welfare ++</th><th>Y/N:</th><th>Welfare +++</th><th>Y/N:</th></tr>
<tr><td>+ When selecting or breeding own replacement animals (heifers and/or bulls) or selecting sires, are undesirable traits recognised and avoided, to improve herd health and welfare? e.g. sire/breed calving ease (to avoid caesareans), calf vigour, pelvic measurements and calm temperament [5]</td><td></td><td>++ Are breeding choices for future herd health and welfare (e.g. avoidance of double muscling) valued equally to breeding choices made for growth rates and other production factors? [5]</td><td></td><td>+++ When selecting replacements are choices made for future long-term improvement of herd health and welfare and resilience valued more than breeding choices made for growth rate and other production factors? [5]</td><td></td></tr>
<tr><td>+ If own replacements are not home bred (store cattle, heifers, cows, bulls, AI sires) is feedback given to the rearers/ breeders/ breed societies/ AI companies as to what traits are important when purchasing livestock or AI straws? [5]</td><td></td><td>++ If surrogate dams are used as recipients for embryos, the breed/breed mix, calving ease scores of the resulting calves is not likely to result in caesarean births? [5]</td><td></td><td>+++ Is data provided to breeding companies/breed societies to improve genetic evaluations and provide other beef farmers the opportunity to select cattle best suited to their own production system/ resources? [5]</td><td></td></tr>
<tr><td colspan="6">Any comments or feedback on this opportunity:</td></tr>
</table>

Good Life Opportunity **Healthy Life** By promoting a natural body type					
**Objective** Cattle should be able to live a life without changing their nature					
**Welfare +**	**Y/N:**	**Welfare ++**	**Y/N:**	**Welfare +++**	**Y/N:**
+ Dehorning (removal of a grown horn) is not undertaken on the farm – is this true?		++ Disbudding (removal of horn bud) is not carried out - is this true?		+++ Neither dehorning/disbudding nor castration are carried out on the farm – is this true? [5]	
+ Branding is not used on the farm as a method of identification– is this true? [24]		**Any comments or feedback on this opportunity:**			
+If castration and/or disbudding (removal of horn buds) is undertaken is anaesthetic AND long-term pain relief used for all ages of cattle including calves less than 2 months old? [5,24,25]					
+ If castration is carried out, is it only done at a young age i.e. calves not adults – is this true? [5]					
+ Neither rubber ring or bloodless (burdizzo) castrations are carried out – is this true? [5]					

References

1. Lundmark Hedman, F.; Hultgren, J.; Röcklinsberg, H.; Wahlberg, B.; Berg, C. Non-Compliance and Follow-Up in Swedish Official and Private Animal Welfare Control of Dairy Cows. Animals 2018, 8, 72.

2. Popescu, S.; Borda, C.; Diugan, E.A.; Spinu, M.; Groza, I.S.; Sandru, C.D. Dairy cows welfare quality in tie-stall housing system with or without access to exercise. *Acta veterinaria Scandinavica* 2013, *55*, 43.

3. Absmanner, E., Rouha-Mülleder, C., Scharl, T., Leisch, F., & Troxler, J. Effects of different housing systems on the behaviour of beef bulls—An on-farm assessment on Austrian farms. *Applied Animal Behaviour Science*, 2009*, 118*(1-2), 12-19.

4. Elmore, M. R. P., Elischer, M. F., Claeys, M. C., & Pajor, E. A. The effects of different flooring types on the behavior, health, and welfare of finishing beef steers. *Journal of animal science*, 2015, *93*(3), 1258-1266.

5. Farm Animal Welfare Committee. Opinion on the Welfare of Cattle Kept For Beef Production. 2019 Available online: https://assets.publishing.service.gov.uk/government/uploads/system/uploads/attachment_data/file/902524/FAWC_Opinion_on_the_welfare_of_cattle_kept_for_beef_production.pdf Accessed 19th December 2021

6. Brscic, M., Gottardo, F., Tessitore, E., Guzzo, L., Ricci, R., & Cozzi, G. Assessment of welfare of finishing beef cattle kept on different types of floor after short-or long-term housing. *Animal*, 2015, *9*(6), 1053-1058.

7. Ninomiya, S., & Sato, S. Effects of 'Five freedoms' environmental enrichment on the welfare of calves reared indoors. *Animal science journal*, 2009, *80*(3), 347-351.

8. Mader, T. L. Animal welfare concerns for cattle exposed to adverse environmental conditions. *Journal of Animal Science*, 2014, *92*(12), 5319-5324.

9. Arnott, G., Ferris, C. P., & O'Connell, N. E. Welfare of dairy cows in continuously housed and pasture-based production systems. *Animal*, 2017, *11*(2), 261-273.

10. Karvatte, N., Klosowski, E. S., de Almeida, R. G., Mesquita, E. E., de Oliveira, C. C., & Alves, F. V. Shading effect on microclimate and thermal comfort indexes in integrated crop-livestock-forest systems in the Brazilian Midwest. *International journal of biometeorology*, 2016, *60*(12), 1933-1941.

11. Rosselle, L., Permentier, L., Verbeke, G., Driessen, B., & Geers, R. Interactions between climatological variables and sheltering behavior of pastoral beef cattle during sunny weather in a temperate climate. *Journal of Animal Science*, 2013, *91*(2), 943-949.

12. Orihuela, A., & Galina, C. S. Effects of Separation of Cows and Calves on Reproductive Performance and Animal Welfare in Tropical Beef Cattle. *Animals*, 2019, *9*(5), 223.

13. Tucker, C. B., Coetzee, J. F., Stookey, J. M., Thomson, D. U., Grandin, T., & Schwartzkopf-Genswein, K. S. Beef cattle welfare in the USA: identification of priorities for future research. *Animal health research reviews*, 2015, *16*(2), 107-124.

14. Westerath, H. S., Laister, S., Winckler, C., & Knierim, U. Exploration as an indicator of good welfare in beef bulls: an attempt to develop a test for on-farm assessment. *Applied Animal Behaviour Science*, 2009, *116*(2-4), 126-133.

15. Ishiwata, T., Uetake, K., Abe, N., Eguchi, Y., & Tanaka, T. Effects of an environmental enrichment using a drum can on behavioral, physiological and productive characteristics in fattening beef cattle. *Animal Science Journal*, 2006, *77*(3), 352-362.

16. Boivin, X., Lensink, J., Tallet, C., & Veissier, I. Stockmanship and farm animal welfare. *Animal Welfare*, 2003, *12*(4), 479-492.

17. Ceballos, M. C., Sant'Anna, A. C., Boivin, X., de Oliveira Costa, F., Monique, V. D. L., & da Costa, M. J. P. Impact of good practices of handling training on beef cattle welfare and stockpeople attitudes and behaviors. *Livestock science*, 2018, *216*, 24-31.

18. Coleman, G. J., & Hemsworth, P. H. Training to improve stockperson beliefs and behaviour towards livestock enhances welfare and productivity. *Rev. Sci. Tech*, 2014, *33*, 131-137.

19. Tucker, C. B., Coetzee, J. F., Stookey, J. M., Thomson, D. U., Grandin, T., & Schwartzkopf-Genswein, K. S. Beef cattle welfare in the USA: identification of priorities for future research. *Animal health research reviews*, 2015, *16*(2), 107-124.

20. Ninomiya, S., & Sato, S. Effects of ‘Five freedoms’ environmental enrichment on the welfare of calves reared indoors. *Animal science journal*, 2009, *80*(3), 347-351.

21. Ninomiya, S. Grooming Device Effects on Behaviour and Welfare of Japanese Black Fattening Cattle. *Animals*, 2019, *9*(4), 186.

22. Arnott, G., Ferris, C. P., & O'Connell, N. E. Welfare of dairy cows in continuously housed and pasture-based production systems. *Animal*, 2017, *11*(2), 261-273.

23. Smid, A. M. C., Weary, D. M., Costa, J. H., & von Keyserlingk, M. A. Dairy cow preference for different types of outdoor access. *Journal of dairy science*, 2018, *101*(2), 1448-1455.

24. Tucker, C. B., Coetzee, J. F., Stookey, J. M., Thomson, D. U., Grandin, T., & Schwartzkopf-Genswein, K. S. Beef cattle welfare in the USA: identification of priorities for future research. *Animal health research reviews*, 2015, *16*(2), 107-124.

25. Canozzi, M. E. A., Mederos, A., Turner, S., Manteca, X., McManus, C., Menegassi, S. R. O., & Barcellos, J. O. J. Dehorning and welfare indicators in beef cattle: a meta-analysis. *Animal Production Science*, 2019, *59*(5), 801-814.
