# Supplementary material for: Advancing a “Good Life” for Farm Animals: Development of Resource Tier Frameworks for On-Farm Assessment of Positive Welfare for Beef Cattle, Broiler Chicken and Pigs
Source: Animals (Basel). 2022 Feb 23;12(5):565. doi: 10.3390/ani12050565 (PMC8908822; doi:10.3390/ani12050565)
Supplement: Supplementary file 1 [file animals-12-00565-s001.zip › S2 Broiler Good Life Framework pre pilot.pdf]

Table S2. Broiler chicken Good Life Framework resulting from the literature review and expert stakeholder feedback which was used in the piloting exercise.

Good life framework – please answer each of the questions with Y (YES) or N (NO) (or N/A if question is not applicable)

Good Life Opportunity Comfort By choice of physical environment					
**Objective** Birds should be able to exercise individual preferences for their physical comfort at all times.					
**Welfare +**	**Y/N:**	**Welfare + +**	**Y/N:**	**Welfare + + +**	**Y/N:**
+ Are birds given a choice of two or more types of suitable litter? (e.g. wood-based litter, peat substitute, straw, sand or equivalent) [1-5].		++ Is there provision of perches and use of slow-growing strain/breed that are better able to utilise perches? [9,6]		+++ Is there a choice of at least two perch types including different heights, diameters, shapes or materials? [6,10,14]	
+ Are birds given elevated resting areas in the form of flat raised platforms (with a ramp for access if fast-growing strains are used)? [6-9]		++ Do perches allow birds to be secure (i.e. allow tendon locking)?		+++ Is there sufficient perch space for all birds to have the choice to rest elevated and undisturbed?	
+ Is there sufficient elevated space to allow 30% of birds undisturbed rest at any one time? [10]		++ Do perches provide at least 15cm perch length per bird? [10,11]		+++ Is there suitable litter to a depth of >10cm?	
		++ Are perches horizontal? [12,13]			
**Any comments or feedback on this opportunity:**					

<table>
<tr><td colspan="6">Good Life Opportunity <u>Comfort</u> <u>By choice of thermal environment</u></td></tr>
<tr><td colspan="6"><u>Objective</u> Birds should be able to exercise individual preferences for their thermal comfort at all times.</td></tr>
<tr><th>Welfare +</th><th>Y/N:</th><th>Welfare + +</th><th>Y/N:</th><th>Welfare + + +</th><th>Y/N:</th></tr>
<tr><td>+ Is there a choice of indoor temperatures i.e. gradient of suitable temperatures within the house?</td><td></td><td>++ Is there access to outdoors to add a choice of temperatures?</td><td></td><td rowspan="4">+++ Are there pop hole rooves and/or cover from wind and rain outside near to pop holes? e.g. veranda, shelters.</td><td rowspan="4"></td></tr>
<tr><td>+ Is there protection from draughts in resting/perching area?</td><td></td><td rowspan="3">++ Is there shade and windbreaks throughout the range as protection from weather?</td><td rowspan="3"></td></tr>
<tr><td>+ Is there access to perches/platforms to improve opportunities for thermoregulation? [6]</td><td></td></tr>
<tr><td>+ Is roof insulation used? e.g. newer shed built with roof insulation [15]</td><td></td></tr>
<tr><td colspan="6">Any comments or feedback on this opportunity:</td></tr>
</table>

Good Life Opportunity Comfort By provision of a safe environment					
**Objective** Birds should be able to exercise individual preferences within their environment with minimum risk of harm<br>NB this section was removed for the purpose of the piloting exercise					
**Welfare +**	**Y/N:**	**Welfare ++**	**Y/N:**	**Welfare +++**	**Y/N:**
+ Are perches more than 8cm but less than 1m above ground level? [16-18]		++ Is consideration given to pop hole height and accessibility for outdoor access?		+++ Is there a policy for monitoring and acting on perch use and incidence of bone fractures e.g. by modifying perch design and layout?	
+ Do perches have a diameter of at least 45mm? [6]					
+ Are perches constructed of/coated in something that enables birds to grip? e.g. wood, rubber [6]		**Any comments or feedback on this opportunity:**			
+ Is there adequate lighting around perches?					
+ Is the angle between perches at different heights, less than 45 degrees? [16,19]					
+ Where horizontal aerial perches are placed in groups, are perches greater than 30cm but less than 1m apart? [16]					

Good Life Opportunity <u>Pleasure by food enrichment</u>					
<u>Objective</u> Birds should be able to exercise individual preferences for food and how it is obtained.					
**Welfare +**	**Y/N:**	**Welfare ++**	**Y/N:**	**Welfare +++**	**Y/N:**
+ Does diet include some food scattered at least once a day? [6]		++ Are wholegrain and/or insects scatter fed at least once a week? [20]		+++ Is there an even distribution (to avoid competition) of forage crops (e.g. brassicas, grass, clover, peas, vetch, lupins, quinoa) available? [6,25,26]	
+ Is insoluble grit provided separately?		++ Is food also offered from a foraging device? (e.g. pecking block, hanging object such as wire bird suet feeder filled with food, strung-up vegetable, maize cobs). [21-24]		+++ Are 'puzzle feeders' (e.g. food ball) provided to prolong provision of food enrichment? [27]	
**Any comments or feedback on this opportunity:**					

Good Life Opportunity **Pleasure** By play				
**Objective Birds should be able to exercise individual preferences for play**				
**Welfare +**	**Welfare ++**	**Y/N:**	**Welfare +++**	**Y/N:**
NO QUESTION IN THIS TIER	++ Are slow growing strains/breeds used, so that birds are more capable of being active and therefore carrying out play behaviour?		+++ Is sufficient space provided throughout the growing period to allow birds to perform play behaviours such as sparring (play-fight), jumping, running with wing-flapping? [28]	
			+++ Are novel objects provided to encourage play behaviour e.g. hanging items, forage blocks/bales?	
**Any comments or feedback on this opportunity:**				

**Good Life opportunity pleasure** By breeding and nurturing experiences					
**Objective Birds should be able to have positive reproductive and nurturing experiences**					
**Welfare +**	**Y/N:**	**Welfare ++**	**Y/N:**	**Welfare +++**	**Y/N:**
+ Are dark brooders used for chicks?		++ Is there provision of adult hens for chicks and pullets?		+++ Are broiler chicks allowed to imprint on adult hens?	
**Any comments or feedback on this opportunity:**					

Good Life Opportunity <u>Confidence</u> <u>By positive experience with stock keepers</u>

<u>Objective</u> Birds should be able to have positive experiences of people when encountered

Welfare +	Y/N:	Welfare ++	Y/N:	Welfare +++	Y/N:
+ Are stock keepers calm (e.g. slow movements) and quiet around the birds? [29]		++ Is specific time (e.g. 15 mins) dedicated to positive interactions on a regular basis (e.g. 3 times per week) from hatching or arrival as day old chicks?		+++ Are positive attitudes towards chickens developed through cognitive behavioural training, including understanding of chicken sensitivity to negative behaviours, e.g. low-stress stockhandling, poultry passport? [29,38]	
+ Do birds experience different people, in different clothes, taking different routes around house on a regular basis?					
+ Is handling gentle, with birds caught and handled in an upright position? [30]		++ Is there provision of enrichment such as platforms and dust baths to reduce fearfulness? [31]		+++ Is more time (>15 mins) dedicated to positive interactions, by more than 1 person, from hatching or day old chicks, on a more regular basis (> 3 times a week)?	
+ Are all stock keepers thoroughly trained on handling and catching OR is mechanical catching used with all stock keepers or contract staff thoroughly trained on use of the machine? [31,32]		**Any comments or feedback on this opportunity:**		+++ Is there an emphasis on good stockmanship even with development of automated technologies such as auto weighing, computer controls. [39]	
+ Is thinning prohibited? [33]				+++ Are there efforts to improve the predictability/controllability for birds by signalling stressful events e.g. knocking on the door before entering?	
+ Is there the promotion of a positive human-animal relationship by associating the presence of humans with non-aversive interactions e.g. talking to birds, maintaining regular visual contact, gentle touching, as well as positive interactions e.g. feeding from hand, whenever in the presence of birds doing routine tasks e.g. feeding and cleaning? [34-37]					

Good Life Opportunity **Confidence** By positive social interactions					
**Objective** Birds should be able to have positive social experiences within their group.					
**Welfare +**	**Y/N:**	**Welfare ++**	**Y/N:**	**Welfare +++**	**Y/N:**
+ Are all resources (food, water, enrichment) spread out evenly to avoid competition between birds?		++ Are there visual barriers (e.g. bales of plastic wrapped wood shavings) vertical panels and/or raised platforms to allow birds to avoid negative social interactions and create the perception of smaller group sizes? [6, 40]		+++ Are there small flock sizes to enable individual recognition and a stable hierarchy? [41]	
+ Does stocking rate allow space to enable birds to avoid antagonistic interactions?		++ Are there fewer, longer pop holes rather than lots of smaller ones?			
		++ Is there provision of a range of perch types (in conjunction with the use of slow-growing strain/breed that can utilise perches? [6]			
**Any comments or feedback on this opportunity:**					

Good Life Opportunity **Interest** By a positively enriched environment					
**Objective** Birds should have opportunities to explore and play in an enriched environment throughout their lives.					
**Welfare +**	**Y/N:**	**Welfare ++**	**Y/N:**	**Welfare +++**	**Y/N:**
+ Are 1 or more types of foraging substrate provided e.g. wood-based litter, peat substitute, straw, sand, oat hulls/husks, soil?		++ Are 2 or more types of foraging substrate provided? [6]		+++ Is daily access given to natural structures (e.g. log piles, fallen down trees on the range, branches, hay bales) or artificial ones (e.g. string, plastic balls, bottles, toys, mirrors) both outdoor and indoors? [52,53]	
+ Is there an even distribution of at least 1 type of foraging item (breeze blocks, forage/lucerne blocks, chopped carrots, nets with cut straw/hay, bale of wood shavings) and 1 manipulation item (e.g. hanging items, CDs, stationary bunches of string/baler twine, spherical objects) changed in form or presentation weekly? [6,22-24,26,42-48]		++ Is there an even distribution of more than 1 type of foraging and manipulation items? [6,50,51]		+++ Are birds provided with outdoor access, in combination with the use of slow-growing strains/ breeds? [6,54,55]	
+ Is there reduced stocking density to allow for increased opportunity to forage? [49]		++ Are there vertical panels and/or barriers (e.g. wooden beams, wood shaving/straw bales) to increase activity, space usage and allow perching and resting? [6]		+++ Do birds have outdoor access before 21 days of age?	
**Any comments or feedback on this opportunity:**					

Good Life Opportunity Interest by enhanced learning opportunities					
**Objective** Birds should be able to experience positive emotional states through cognitive enrichment.					
**Welfare +**	**Y/N:**	**Welfare ++**	**Y/N:**	**Welfare +++**	**Y/N:**
+ Is the complexity of the environment increased to stimulate learning e.g. provision of straw bales, different types of vegetation?		++ Is daily access (at least 4 hours per day) to range given? (In combination with use of slow-growing strains/breeds)		+++ Is daily access given to learning enrichments e.g. mazes, branches, even distribution of feeding devices and tasks such as 'puzzle feeders' e.g. food ball? (only in combination with use of slow-growing strains/breeds). [56-58]	
**Any comments or feedback on this opportunity:**					

Good Life Opportunity Interest By promoting ranging

Objective Birds should be able to have positive experiences of the outdoor environment.

Welfare +	Y/N:	Welfare ++	Y/N:	Welfare +++	Y/N:
+ Is daily access given to a well-drained range with covered structures e.g. shade and shelter panels and hedges/shrubs? [6,41,59,60]		++ Is shelter/cover provided in the form of dense vegetation, including trees? [6]		+++ Is there substantial woodland/forest area (at least 25% of range area) for ranging including some mature trees (>2yrs)?	
+ Are covered structures and hedges/shrubs visible from the pop holes and distributed evenly throughout the range?		++ Are there covered dustbathing opportunities e.g. roofed sandpit distributed evenly on the range?		+++ Is there access to edible vegetation e.g. grass, clover, chicory, either scattered or growing on the range?	
+ Do covered structures and vegetation start no further than 3m from the pop holes?		++ Do covered dustbathing opportunities start no further than 10m from the pop hole?			
+ Are slow-growing strains/breeds used? [6]		++ Are there other animals (ruminants) using the range either at the same time as the birds or at different times?			

Any comments or feedback on this opportunity:

Good Life Opportunity Healthy Life By dustbathing choices					
**Objective** Birds should be exercise individual preferences for dustbathing substrate and location					
**Welfare +**	**Y/N:**	**Welfare ++**	**Y/N:**	**Welfare +++**	**Y/N:**
+ Is stocking density sufficient to allow space for dustbathing?		+ Are slow growing breeds/ strains used?		+++ Is there a choice of more than 1 dustbathing substrates in outdoor shelter/veranda?	
+ Is there continuous access to areas containing dustbathing substrate (e.g. wood-based litter, peat substitute, straw, sand, oat hulls/husks or equivalent)? [1-6,9]		++ Is there access to covered dustbathing areas (e.g. roofed sandpit) outdoors or in a veranda?		+++ Is the litter friable and deeper than 10 cm?	
		++ Are there daylight-type bulbs in housing, if artificial lighting is used?			
**Any comments or feedback on this opportunity:**					

Good Life opportunity Healthy life By resting choices					
**Objective** Birds should have opportunities to rest undisturbed					
**Welfare +**	**Y/N:**	**Welfare ++**	**Y/N:**	**Welfare +++**	**Y/N:**
+ Do lighting patterns allow synchronised behaviour (undisturbed rest) including a minimum 6 hours uninterrupted dark? [61,62]		++ Are perches and/or platforms provided to reduce disturbances and so allow undisturbed rest? [6]		+++ Are panels and/or barriers (e.g. wooden beams, straw or wood shaving bales) provided to reduce disturbances and so allow undisturbed rest? [6,9]	
+ Is natural light provided? i.e. at least 20 lux covering 3% of the floor. [9]				+++ Are birds given the choice of when to rest and when to be active if artificial lighting is used, by using "dual light intensity choice" e.g. providing a light intensity of 2 lux in one area/compartment of the shed and 20 lux in another? [63]	
+ Is litter maintained in a dry and friable condition? [62]					

Any comments or feedback on this opportunity:

Good Life Opportunity Healthy Life by management policy for positive health					
**Objective** Stock keepers should manage day to day health effectively					
**Welfare +**	**Y/N:**	**Welfare ++**	**Y/N:**	**Welfare +++**	**Y/N:**
+ Is a health and welfare programme implemented and reviewed frequently plus action taken to reduce or alleviate the cause of any health and welfare problems?		++ Is there regular dialogue (at least once per crop) with vet and/or scheme welfare advisor?		+++ Do farm staff take active part in welfare activities with wider benefits (e.g. member of scheme policy/ management group, peer advisor, on-farm welfare research)?	
+ Medicines are not used on a routine basis as substitute for good management – is this true?		**Any comments or feedback on this opportunity:**			
+ Thinning is prohibited – is this true? [33]					
+ Are procedures in place to reduce risk of disease in outdoor range as well as predation e.g. access to cover? [33]					
+ Is there a policy for monitoring and culling sick birds?					

**Good Life Opportunity** **Healthy Life** By positive genetic selection for long-term health and welfare					
**Objective** **Stock keepers should influence long term health and welfare of birds.**					
**Welfare +**	**Y/N:**	**Welfare +**	**Y/N:**	**Welfare +**	**Y/N:**
+ Does the farm manager recognise undesirable side-effects of genetic selection for production efficiency and choose replacement birds to reduce/mitigate for current health and welfare problems within flock (e.g. lameness, sudden death syndrome, ascites) i.e. choose slow growing strains/ breeds over fast growing ones, where the farm manager has control over choice of replacements? [6,11,64-68]		++ Are breed/bird choices, made to mitigate potential issues for future flock health and welfare, valued **equally** to choices made for growth rate and other production factors?		+++ Are replacements chosen for long term improvement of flock health and welfare, resilience and metabolic normality, valuing these **more** than choices made for growth rate and other production factors?	
+ If own replacements are not selected/bred by the unit manager is feedback given to the breeders/genetic companies/hatcheries which supply the chicks, as to what traits are important to the manager?					
**Any comments or feedback on this opportunity:**					

References

1. Scholz, B., Urselmans, S., Kjaer, J.B., Schrader, L .2010. Food, wood, or plastic as substrates for dustbathing and foraging in laying hens: A preference test. Poultry Science, 89, 1584-1589.

2. de Jong, I.C., Fillerup, M., van Reenen, K. 2005. Substrate preferences in laying hens. Animal Science Papers and Reports, 23, 143-151.

3. de Jong, I.C., Wolthuis-Fillerup, M., van Reenen, C.G. 2007. Strength of preference for dustbathing and foraging substrates in laying hens. Applied Animal Behaviour Science, 104, 24-36.

4. Wichman, A., Keeling. L.J. 2008. Hens are motivated to dustbathe in peat irrespective of being reared with or without a suitable dustbathing substrate. Animal Behaviour, 75, 1525-1533.

5. Gunnarsson, S., Keeling, L. J., Svedberg, J. 1999. Effect of rearing factors on the prevalence of floor eggs, cloacal cannibalism and feather pecking in commercial flocks of loose housed laying hens. British Poultry Science, 40, 12-18.

6. Riber, A. B., de Jong, I. C., van de Weerd, H. A., & Steenfeldt, S. (2017). Environmental enrichment for broiler breeders: An undeveloped field. *Frontiers in veterinary science*, *4*, 86.

7. Bailie, C. L., Baxter, M., & O'Connell, N. E. (2018). Exploring perch provision options for commercial broiler chickens. *Applied animal behaviour science*, *200*, 114-122.

8. Norring, M., Kaukonen, E., & Valros, A. (2016). The use of perches and platforms by broiler chickens. *Applied Animal Behaviour Science*, *184*, 91-96..

9. de Jong, I. C., & Gunnink, H. (2019). Effects of a commercial broiler enrichment programme with or without natural light on behaviour and other welfare indicators. *animal*, *13*(2), 384-391.

10. Malchow, J., Berk, J., Puppe, B., & Schrader, L. (2018). Perches or grids? What do rearing chickens differing in growth performance prefer for roosting?. *Poultry science*, *98*(1), 29-38.

11. Gebhardt-Henrich, S. G., Toscano, M. J., & Würbel, H. (2017). Perch use by broiler breeders and its implication on health and production. *Poultry science*, *96*(10), 3539-3549.

12. LeVan, N. F., Estevez, I., & Stricklin, W. R. (2000). Use of horizontal and angled perches by broiler chickens. *Applied Animal Behaviour Science*, *65*(4), 349-365.

13. Pettit-Riley, R., & Estevez, I. (2001). Effects of density on perching behavior of broiler chickens. *Applied Animal Behaviour Science*, *71*(2), 127-140.

14. Jones, R.B., Carmichael, N.L., Rayner, E. 2000. Pecking preferences and pre-dispositions in domestic chicks: implications for the development of environmental enrichment devices. Applied Animal Behaviour Science, 69, 291-312.

15. Baracho, M. S., Nääs, I. A., Betin, P. S., & Moura, D. J. (2018). Factors that Influence the Production, Environment, and Welfare of Broiler Chicken: A Systematic Review. *Brazilian Journal of Poultry Science*, *20*(3), 617-624.

16. Struelens, E., & Tuyttens, F. A. M. (2009). Effects of perch design on behaviour and health of laying hens. *Animal Welfare*, *18*(4), 533-538.

17. Sandilands, V., Moinard, C., Sparks, N.H.C. 2009. Providing laying hens with perches: fulfilling behavioural needs but causing injury? British Poultry Science, 50, 395-406.

18. Schrader, L., Muller, B. 2009. Night-time roosting in the domestic fowl: The height matters. Applied Animal Behaviour Science, 121, 179-183.

19. Scott, G.B., Lambe, N.R., Hitchcock, D. 1997. Ability of laying hens to negotiate horizontal perches at different heights, separated by different angles. British Poultry Science, 38, 48-54.

20. Rose, S.P., Fielden, M., Foote, W.R., Gardin, P. 1995. Sequential feeding of whole wheat to growing broiler chickens. British Poultry Science, 36, 97-111.

21. Aerni, V., El-Lethey, H., Wechsler, B. 2000. Effect of foraging material and food form on feather pecking in laying hens. British Poultry Science, 41, 16-21.

22. El-Lethey, H., Aerni, V., Jungi, T.W., Wechsler, B. 2000. Stress and feather pecking in laying hens in relation to housing conditions. British Poultry Science, 41, 22-28.

23. Dixon, L. M., Duncan, I. J. H., Mason, G.J. 2010. The effects of four types of enrichment on feather-pecking behaviour in laying hens housed in barren environments. Animal Welfare, 19, 429-435.

24. Hartini, S., Choct, M., Hinch, G., Kocher, A., Nolan, J. V. 2002. Effects of light intensity during rearing and beak trimming and dietary fiber sources on mortality, egg production, and performance of ISA brown laying hens. Journal of Applied Poultry Research, 11, 104-110.

25. Horsted, K., Hermansen, J.E. 2007. Whole wheat versus mixed layer diet as supplementary feed to layers foraging a sequence of different forage crops. Animal, 1, 575-585.

26. Steenfeldt, S., Kjaer, J. B., Engberg, R. M. 2007. Effect of feeding silages or carrots as supplements to laying hens on production performance, nutrient digestibility, gut structure, gut microflora and feather pecking behaviour. British Poultry Science, 48, 454-468.

27. Pichova, K., Nordgreen, J., Leterrier, C., Kostal, L., & Moe, R. O. (2016). The effects of food-related environmental complexity on litter directed behaviour, fear and exploration of novel stimuli in young broiler chickens. *Applied Animal Behaviour Science*, *174*, 83-89.

28. Baxter, M., Bailie, C. L., & O'Connell, N. E. (2019). Play behaviour, fear responses and activity levels in commercial broiler chickens provided with preferred environmental enrichments. *animal*, *13*(1), 171-179.

29. Coleman, G. J., & Hemsworth, P. H. (2014). Training to improve stockperson beliefs and behaviour towards livestock enhances welfare and productivity. *Rev. Sci. Tech*, *33*, 131-137.

30. Kittelsen, K., Granquist, E., Aunsmo, A., Moe, R., & Tolo, E. (2018). An evaluation of two different broiler catching methods. *Animals*, *8*(8), 141.

31. Pilecco, M., Almeida Paz, I. C. L., Tabaldi, L. A., Nääs, I. A., Garcia, R. G., Caldara, F. R., & Francisco, N. S. (2013). Training of catching teams and reduction of back scratches in broilers. *Brazilian Journal of Poultry Science*, *15*(3), 283-286.

32. de Jong, I., Berg, C., Butterworth, A., & Estevéz, I. (2012). Scientific report updating the EFSA opinions on the welfare of broilers and broiler breeders. *EFSA Supporting Publications*, *9*(6), 295E.

33. Higham, L. E., Scott, C., Akehurst, K., Dring, D., Parnham, A., Waterman, M., & Bright, A. (2018). Effects of financial incentives and cessation of thinning on prevalence of Campylobacter: a longitudinal monitoring study on commercial broiler farms in the UK. *Veterinary Record*, vetrec-2017.

34. Barnett, J.L., Hemsworth, P.H., Hennessy, D.P., McCallum, T.H., Newman, E.A. 1994. The effects of modifying the amount of human contact on behavioural, physiological, physiological responses of laying hens. Applied Animal Behaviour Science, 41, 87-100.

35. Gross, W.B., Siegel, P.B. 1982. Influences of sequences of environmental factors on the response of chickens to fasting and to staphyloccus aureous infection. American Journal of Veterinary Research, 43, 137-139.

36. Gross, W.B., Siegel, P.B. 1980. Effects of early life environmental stresses on chicken body weight, antibody response to RBC antigens, feed efficiency and response to fasting. Avian Diseases, 24, 569-579.

37. Gross, W.B., Siegel, P.B. 1979. Adaptation of chickens to their handler, and experimental results. Avian Diseases, 23, 708-714.

38. Reported in: Boivin, X., & Ruis, M. A. W. (2011). " Quality Handling" a training program to reduce fear and stress in farm animals. In *4th Boehringer Ingelheim Expert Forum on farm animal well-being: May 27th 2011, Seville (Spain)* (pp. 20-27).

39. Dawkins, M. S., Donnelly, C. A., & Jones, T. A. (2004). Chicken welfare is influenced more by housing conditions than by stocking density. *Nature*, *427*(6972), 342.

40. Friere, R., Wilkins, L.J., Short, F., Nicol, C.J. 2003. Behaviour and welfare of individual hens in a non- cage system. British Poultry Science, 44, 22-29.

41. Bestman, M.W.P., Wagenaar, J.P. 2003. Farm level factors associated with feather pecking in organic laying hens. Livestock Production Science, 80, 133-140.

42. Holcman, A., Gorjanc, G., Stuhec, I. 2008. Porous concrete block as an environmental enrichment device increases activity of laying hens in cages. Poultry Science, 87, 1714-1719.

43. Jones, R.B. 2004. Environmental enrichment: the need for practical strategies to improve poultry welfare. In Welfare of the Laying Hen. Perry ,G. (Ed). pp215-225. CABI Publishing, Cambridge.

44. Jones, R.B., Carmichael, N.L., Rayner, E. 2000. Pecking preferences and pre-dispositions in domestic chicks: implications for the development of environmental enrichment devices. Applied Animal Behaviour Science, 69, 291-312.

45. Jones, R.B. 2001. Does occasional movement make pecking devices more attractive to domestic chicks? British Poultry Science, 42, 43-50.

46. McAdie, T.M, Keeling, L. J., Blokhuis, H.J., Jones, R.B. 2005. Reduction in feather pecking and improvement of feather condition with the presentation of a string device to chickens. Applied Animal Behaviour Science, 93, 67-80.

47. Gvaryahu, G., Ararat, E., Asaf, F.E., Lev, S.M., Weller, J.I, Robinzon, B. Snapir, N. 1994. An enrichment object that reduces aggressiveness and mortality in caged laying hens. Physiology & Behavior, 55, 313-316.

48. Sherwin, C.M. 1995. Environmental enrichment for laying hens: Spherical objects in the feed trough. Animal Welfare, 4, 41-51.

49. Tahamtani, F. M., Pedersen, I. J., Toinon, C., & Riber, A. B. (2018). Effects of environmental complexity on fearfulness and learning ability in fast growing broiler chickens. *Applied animal behaviour science*, *207*, 49-56.

50. Chow, A., Hogan, J. A. 2005. The development of feather pecking in Burmese red junglefowl: the influence of early experience with exploratory-rich environments. Applied Animal Behaviour Science, 93, 283-294.

51. Huber-Eicher, B., Sebö, F. 2001. Reducing feather pecking when raising laying hen chicks in aviary systems. Applied Animal Behaviour Science, 73, 59–68.

52. Jones, R.B., Waddington, D. 1992. Modification of fear in domestic chicks, Gallus gallus domesticus, via regular handling and early environmental enrichment. Animal Behaviour, 4,: 1021-1033.

53. Jones, R.B. 1996. Fear and adaptability in poultry: Insights, implications and imperatives. Worlds Poultry Science Journal, 52, 131-174.

54. Zhao, Z. G., Li, J. H., Li, X., & Bao, J. (2014). Effects of housing systems on behaviour, performance and welfare of fast-growing broilers. *Asian-Australasian journal of animal sciences*, *27*(1), 140.

55. Taylor, P. S., Hemsworth, P. H., Groves, P. J., Gebhardt-Henrich, S. G., & Rault, J. L. (2019). Frequent range visits further from the shed relate positively to free-range broiler chicken welfare. *animal*, 1-12.

56. Manteuffel, G., Langbein, J., Puppe, B. 2009. Increasing farm animal welfare by positively motivated instrumental behaviour. Applied Animal Behaviour Science, 118, 191-198.

57. Reported in: Osborne, S. R. (1977). The free food (contrafreeloading) phenomenon: A review and analysis. *Animal Learning & Behavior*, *5*(3), 221-235.

58. Lindqvist, C., Zimmerman, P., & Jensen, P. (2006). A note on contrafreeloading in broilers compared to layer chicks. *Applied Animal Behaviour Science*, *101*(1-2), 161-166.

59. Nicol, C. J., Pötzsch, C., Lewis, K., Green, L. E. 2003. Matched concurrent case-control study of risk factors for feather pecking in hens on free-range commercial farms in the UK. British Poultry Science, 44, 515-523.

60. Zeltner, E., Hirt, H. 2008. Factors involved in the improvement of the use of hen runs. Applied Animal Behaviour Science, 114, 395-408.

61. Karaarslan, S., & Nazlıgül, A. (2018). Effects of lighting, stocking density, and access to perches on leg health variables as welfare indicators in broiler chickens. *Livestock science*, *218*, 31-36.

62. Bassler, A. W., Arnould, C., Butterworth, A., Colin, L., De Jong, I. C., Ferrante, V., ... & Blokhuis, H. J. (2013). Potential risk factors associated with contact dermatitis, lameness, negative emotional state, and fear of humans in broiler chicken flocks. *Poultry Science*, *92*(11), 2811-2826.

63. Kang, S. W., Christensen, K. D., Aldridge, D., & Kuenzel, W. J. (2020). Effects of light intensity and dual light intensity choice on plasma corticosterone, central serotonergic and dopaminergic activities in birds, Gallus gallus. *General and comparative endocrinology*, *285*, 113289.

64. Zuidhof, M. J., Schneider, B. L., Carney, V. L., Korver, D. R., & Robinson, F. E. (2014). Growth, efficiency, and yield of commercial broilers from 1957, 1978, and 2005. *Poultry Science*, *93*(12), 2970-2982.

65. Bessei, W. (2006). Welfare of broilers: a review. *World's Poultry Science Journal*, *62*(3), 455-466.

66. Knowles, T. G., Kestin, S. C., Haslam, S. M., Brown, S. N., Green, L. E., Butterworth, A., ... & Nicol, C. J. (2008). Leg disorders in broiler chickens: prevalence, risk factors and prevention. *PloS one*, *3*(2), e1545.

67. McGeown, D., Danbury, T. C., Waterman-Pearson, A. E., & Kestin, S. C. (1999). Effect of carprofen on lameness in broiler chickens. *Veterinary Record*, *144*(24), 668-671.

68. Danbury, T. C., Weeks, C. A., Waterman-Pearson, A. E., Kestin, S. C., & Chambers, J. P. (2000). Self-selection of the analgesic drug carprofen by lame broiler chickens. *Veterinary Record*, *146*(11), 307-311.
