# Supplementary material for: Advancing a “Good Life” for Farm Animals: Development of Resource Tier Frameworks for On-Farm Assessment of Positive Welfare for Beef Cattle, Broiler Chicken and Pigs
Source: Animals (Basel). 2022 Feb 23;12(5):565. doi: 10.3390/ani12050565 (PMC8908822; doi:10.3390/ani12050565)
Supplement: Supplementary file 1 [file animals-12-00565-s001.zip › S3 Pig Good Life Framework pre pilot.pdf]

Table S3. Pig Good Life Framework resulting from the literature review and expert stakeholder feedback which was used in the piloting exercise.

Good life framework – please answer each of the questions with Y (YES) or N (NO) (or N/A if question is not applicable)

**Good Life Opportunity Comfort By choice of physical environment**					
**Objective Pigs should be able to exercise individual preferences for their physical comfort at all times as follows:**					
**Welfare +**	**Y/N:**	**Welfare ++**	**Y/N:**	**Welfare ++++**	**Y/N:**
+ Is the lying area of solid construction?		++ Are at least 2 different types of bedding provided? e.g. straw, sawdust, rice husks, a peat substitute, sand. [1,2]		+++ Is there more space than needed for all pigs to lie down simultaneously, to allow choice of where to lie?	
+ Is bedding [1] to a depth <10cm provided?		++ Is bedding to a depth of >10cm?			
**Any comments or feedback on this opportunity:**					

Good Life Opportunity Comfort By choice of thermal environment					
**Objective** Pigs should be able to exercise individual preferences for their thermal comfort at all times.					
**Welfare +**	**Y/N:**	**Welfare ++**	**Y/N:**	**Welfare +++**	**Y/N:**
+ Do pigs have a choice of indoor temperatures e.g. access to area of deeper bedding or area of bare concrete floor? [1]		++ Do pigs have the opportunity to spend at least part of the day/night outdoors with access to shade?		+++ Is there enough space in wallows for all pigs to use at the same time?	
+ Is there enough space for pigs to choose which thermal environment to be in?		++ Do pigs have the opportunity to access a (non-faecal) wallow (outdoors and/or indoors e.g. water pit)? [3]		+++ Is there enough space for all pigs to access shade at the same time?	
**Any comments or feedback on this opportunity:**					

Good Life Opportunity Pleasure by food enrichment					
**Objective** Pigs should be able to exercise individual preferences for type of food and how it is obtained.					
**Welfare +**	**Y/N:**	**Welfare ++**	**Y/N:**	**Welfare +++**	**Y/N:**
+ Do pigs receive roughage such as silage, available clean each day? [4,5]		++ Do pigs receive part of diet scattered widely on rootable substrate daily? [5]		+++ Do pigs receive other forms of ingestible enrichment e.g. peanuts/straw/hay/vegetables in box, hanging vegetables, foraging balls? [6-8]	
+ Do pigs receive part of their diet scattered widely on a clean floor daily?					
**Any comments or feedback on this opportunity:**					

Good Life Opportunity <u>Pleasure</u> <u>By play</u>

<u>Objective</u> Pigs should be able to experience individual experiences for play

**Welfare +**	**Y/N:**	**Welfare ++**	**Y/N:**	**Welfare +++**	**Y/N:**
+ Do pigs have permanent access to rootable substrate (straw, hay, wood, sawdust, mushroom compost, peat substitute or a mixture)? [9-12]		++ Are there other substrates in addition to straw (e.g. wood bark, branches, large hay bales)? [5,9,10]		+++ Are there at least three clean toys?	
+ Are there at least two clean toys (e.g. soft malleable rubber toys and balls, ropes, plastic piping)? [13-19]		++ Is there sufficient space inside housing to allow running at least 10 paces in one direction during all life stages? [16,18]		+++ Are toys changed on a weekly basis so that the same toy is not presented within at least two months?	
				+++ Are there procedures in place to enable early handling of pigs including stroking? [22]	
+ Are toys changed on a weekly basis so that the same toy is not presented within at least a one month period? [20,21]				+++ Are piglets reared (i.e. from birth to weaning) outdoors? [12]	

Any comments or feedback on this opportunity:

**Good Life Opportunity <u>Pleasure</u>** <u>By breeding and nurturing experiences</u>					
**<u>Objective</u> Pigs should be able to have positive reproductive and nurturing experiences.**					
**Welfare +**	**Y/N:**	**Welfare ++**	**Y/N:**	**Welfare +++**	**Y/N:**
+ Is enough space provided for the sow to turn around during the farrowing period? [23]		++ Is the nesting area enclosed by three solid dark walls? [25]		+++ Is there enough space for a nesting area and an activity area for the sow?	
+ Is nesting material (e.g. long-stemmed straw) provided 48 hours to farrowing? [23]		++ Does the nesting area allow for gradual lying down? [25,26]		+++ Do the piglets have access to the sow for 56 days or more?	
+ Do piglets have access to the sow for 33 - 39 days?		++ Is there sufficient nesting material to build and replenish a nest approximately 2m in diameter?			
+ Is weaning gradual? e.g. social separation from the sow in increasing duration and frequency over time. [24]		++ Is there sufficient space for the sow to be able to move away from the piglets? [27]			
		++ Do piglets have access to the sow for 40 - 55 days?			
**Any comments or feedback on this opportunity:**					

**Good Life Opportunity <u>Confidence</u> <u>By positive experience with stockpersons</u>**					
**<u>Objective</u> Pigs should have positive experiences of people when encountered**					
**Welfare +**	**Y/N:**	**Welfare ++**	**Y/N:**	**Welfare +++**	**Y/N:**
+ Goads are never used on the farm –is this true?		++ Is specific time (e.g. 15 mins) per pen dedicated to positive interactions with at least some of the pigs per pen on a regular basis (e.g. 3 times a week), from birth? [22,31]		+++ Do staff undergo specific training (e.g. ProHand Pigs, Quality Handling) to enable them to develop positive relationships with the pigs? [22]	
+ Is calm speech used around the pigs at all times (no shouting)? [22,28,29]				+++ Is specific time (> 15 mins per pen) dedicated positive interactions for all pigs, on a more regular basis (> 3 times per week) from birth?	
+ Are all handling procedures carried out gently? [22]				+++ Are positive interactions with pigs carried out by more than one person?	
+ Is the presence of people associated with positive events such as offering food, positive interactions such as stroking and scratching, to at least some of the pigs whenever carrying out routine tasks e.g. feeding? [22,28-30]		**Any comments or feedback on this opportunity:**			

<table>
<tr><th colspan="6">Good Life Opportunity <u>Confidence</u> <u>By positive social interactions</u></th></tr>
<tr><th colspan="6"><u>Objective</u> Pigs should be able to have positive social experiences within their group.</th></tr>
<tr><th>Welfare +</th><th>Y/N:</th><th>Welfare ++</th><th>Y/N:</th><th>Welfare +++</th><th>Y/N:</th></tr>
<tr><td>+ If pigs are mixed, are group sizes large (>20 pigs)? [32]</td><td></td><td>++ Are piglets socialised during lactation period (co-mingle with other piglets) after 10 days of age? [32,32]</td><td></td><td>+++ Is any mixing done before weaning, so that pigs are kept in stable groups with no mixing post weaning? [32]</td><td></td></tr>
<tr><td>+ Are smaller groups housed together before introducing into larger groups (allowing pigs to from subgroups)? [32]</td><td></td><td>++ If sows/fattening pigs are mixed, are pigs of same sex and with obviously different weights? [32]</td><td></td><td>+++ Are pigs selected for more positive social behaviour and reduced negative behaviour? [11]</td><td></td></tr>
<tr><td>+ Is there ample space to allow pigs to choose who to spend time with and who to avoid? [32,11]</td><td></td><td>++ If sows/fattening pigs are mixed, is accessibility to resources (e.g. drinkers, lying spaces, enrichment objects) increased in terms of pen layout, number and spread of resources? [32]</td><td></td><td colspan="2" rowspan="5">Any comments or feedback on this opportunity:</td></tr>
<tr><td>+ Are there adequate opportunities to avoid, escape or hide from other pigs (e.g. visual barriers, shape of pen allowing escape opportunities/prevent cornering)? [32]</td><td></td><td>++ If sows/fattening pigs are mixed, are pigs floor-fed rather than in stalls or electric feeders? [32]</td><td></td></tr>
<tr><td>+ If electronic sow feeding is used, is the design as follows: low number of sows per ESF, a long route from exit to entry of the ESF and positioning the ESF away from busy areas or other resources? [32]</td><td></td><td>++ If sows/fattening pigs are mixed, is time spent foraging increased by increasing frequency and amount of feed, with feed/food enrichment spread throughout the pen? [32]</td><td></td></tr>
<tr><td>+ Do number of feeders, feeder holes and spaces allow for avoidance of feeding competition? [32]</td><td></td><td rowspan="2">++ If sows are mixed, is the presence of a boar (in separate housing) provided? [32]</td><td rowspan="2"></td></tr>
<tr><td>+ Is enough extra enrichment provided at point of mixing, to avoid competition? e.g. roughage, silage, sticks, toys, logs etc. [32]</td><td></td></tr>
<tr><th colspan="6">Good Life Opportunity <u>Interest</u> <u>By a positively enriched environment</u></th></tr>
</table>

**Objective** Pigs should have opportunities to explore and play in an enriched environment throughout their lives.					
**Welfare +**	**Y/N:**	**Welfare ++**	**Y/N:**	**Welfare +++**	**Y/N:**
+ Do pigs have access to either 1 rooting substrate plus 1 item of object enrichment, OR 2 rooting substrates? Substrates to allow pigs to root, investigate, chew and play include: straw, a peat substitute, mushroom compost (or other type of compost), soil, sand, sawdust, wood shavings, wood bark, hay, grass. Object enrichment includes hanging objects e.g. hanging branched chain, soft malleable rubber dog toy, rope, plastic ball, plastic piping, soft wood or logs, and non-hanging items e.g. sticks, cardboard boxes, branches, loose logs, wooden planks. [5,9-18,21]		++ Do pigs have access to either at least 2 rooting substrates plus 1 item of object enrichment, OR at least 2 rooting substrates,1 of which must be outdoor grass/ earth?		+++ Is access to outdoor pasture or woodland or root crops provided during daytime? [12,36]	
+ Are any objects in contact with the floor kept clean? [21]		++ Is long straw provided and regularly renewed? [5]		+++ Is access to at least 2 rooting substrates and either at least 2 items of object enrichment OR inclusion of trees and other types vegetation within the pasture provided?	
+ Is object enrichment sufficiently provided and spaced out to enable proper manipulation and avoid fighting? [12]		++ Is object enrichment made of chewable and destructible materials such as ropes, paper, jute? [5,21,34]		+++ Is object enrichment provided in the form of mixed substrates e.g. long straw with fir branches, straw with forest bark and branches, or objects made of a combination of materials, e.g. ropes and rubber hoses, and is it destructible, deformable, complex,	

				changeable and sometimes containing ingestible parts? [5,6,10,37,38]	
+ Is object enrichment changed to different objects on a weekly basis so that the same object/toy is not presented within at least a 1 month period? [14,20,21]		++ Does space inside housing allow opportunities for rooting, exploration and play? [36]		+++ Is there continuous improvement of object enrichment through continuously providing new and various types and selecting the most successful? [21]	
		++ Are straw bales or large hay bales provided? [5]		+++ Are piglets outdoor reared (i.e. from birth to weaning)? [12]	
		++ Is this level of enrichment provided from birth to slaughter? [18]			

Any comments or feedback on this opportunity:

Good Life Opportunity <u>Interest</u> <u>By enhanced learning opportunities</u>

<u>Objective</u> Pigs should be able to experience positive emotional states through cognitive enrichment.

Welfare +	Y/N:	Welfare ++	Y/N:	Welfare +++	Y/N:
+ Is some food provided in 'puzzle feeders' such as food ball, with enough puzzle feeders provided to avoid competition? i.e. if stockpersons see aggression relating to puzzle feeders they know to add another and another until aggression stops. [11,39,40]		++ Are 'puzzle feeders' of increasing difficulty (requiring more manipulation to release food) provided, to maintain cognitive challenge, with enough puzzle feeders provided to avoid competition? [39,40]		+++ Is there a call-feeding station with a program to increase the difficulty (requiring pressing of button/lever, and increasing number of presses required)? [40,42-47]	
		++ Are cues (e.g. sounds) used to signal enrichment events such as the provision of a food ball? [40,41]		+++ Is there a call-feeding station with a program to increase the difficulty (requiring pressing of button/lever, and increasing number of presses required)? [47]	
		++ Are these cues provided at random times (and locations if possible)? [40]			
		++ Is there a delay of a few seconds between a cue and providing the enrichment, for anticipation of positive event? [39-42]			

Any comments or feedback on this opportunity:

Good Life Opportunity Healthy Life by management policy for positive health

<u>Objective</u> Pig stock keepers should manage day to day pig health effectively

Welfare +	Y/N:	Welfare ++	Y/N:	Welfare +++	Y/N:
+ Is a health and welfare programme implemented and reviewed frequently i.e. every 6 months or more often plus action taken to reduce or alleviate the cause of any health and welfare problems?		++ Is there regular dialogue with a vet and/or scheme welfare advisor?		+++ Do stock-keepers manage vulnerable individual animals (e.g. piglets) with attention and positive management strategies for their individual needs?	
+ Is it true that routine use of medicines is not used as a substitute for good management?				+++ Do stock-keepers take active part in welfare activities with wider benefits (e.g. member of scheme policy/ management group, peer advisor, on-farm welfare research)?	
+ Do stock-keepers manage vulnerable groups (e.g. piglets) with attention and positive management strategies for their group needs?					

Any comments or feedback on this opportunity:

Good Life Opportunity Healthy Life By positive genetic selection for long term health and welfare

Objective Unit managers and stock persons should positively influence the long term health and welfare of pigs

Welfare +	Y/N:	Welfare ++	Y/N:	Welfare +++	Y/N:

+ Does the farm manager recognise undesirable side-effects of genetic selection for production efficiency and choose replacement animals to reduce/mitigate for current health and welfare problems within the herd (e.g. piglet mortality, tail biting), where the farm manager has control over choice of replacement animals? [48]		++ Does the farm manager make choices for potential future health and welfare issues within the herd, valuing these equally to litter size, growth rate and other production factors? [48]		+++ Are replacement animals chosen for long-term improvement of herd health and welfare, resilience and metabolic normality, valuing these more over litter size, growth rate and other production factors? e.g. selecting for piglet survival v number born, improved placental efficiency, reduced intra-litter birth weight variability, more robust piglets, improved maternal behaviour, sows selected for specific loose-farrowing traits, smaller litter size or at least ceasing to select for increased litter sizes, breeding for improved social relationships. [27,48-51]	
+ If own replacements are not home bred or selected by the unit manager is feedback given to the breeders/ genetic companies/rearers as to what traits are important to the manager?		++ Do selection criteria include piglet survival in addition to number of piglets born?			
**Any comments or feedback on this opportunity:**					

Good Life opportunity Healthy life By promoting a natural body type

Objective **Pigs should be able to live a life without changing their nature**

Welfare +	Y/N:	Welfare ++	Y/N:	Welfare +++	Y/N:
+ Not all outdoor sows are nose ringed – is this true?		**++** Are the majority (>50%) of sows not nose ringed?		+++ There are no sows on the unit with nose rings – is this true?	
+ If a pig is nose ringed, only 1 ring is inserted and this is not replaced if it falls out – is this true?		**++** Are the majority (>50%) of pigs and piglets not tail docked?		+++ Are no pigs and piglets tail docked?	
+ Are some (any) pigs and piglets not tail docked and long tailed pigs have not received treatment or been culled in the last 3 months?		++ Long tailed pigs have not received treatment or been culled in the last 3 months – is this true?		+++ No piglets have had teeth reduced – is this true?	
+ Are long- and short-tailed pigs are kept in separate groups?		++ Do the majority (>50%) of piglets not have teeth reduced?		+++ No boars are castrated- is this true?	
+ Not all piglets have teeth reduced – is this true?		<u>Question only for NON FARM ASSURED units:</u><br>++ Is chemical castration used?			
+ Are some (any) boars not castrated?					
**Any comments or feedback on this opportunity:**					

References

1. Tuyttens, F. A. M. The importance of straw for pig and cattle welfare: a review. *Applied animal behaviour science*, 2005, *92*(3), 261-282.

2. Hötzel, M. J., Lopes, E. J. C., De Oliveira, P. A. V., & Guidoni, A. L. Behaviour and performance of pigs finished on deep bedding with wood shavings or rice husks in summer. *Animal Welfare*, 2009 *18*(1), 65-71.

3. Cecchin, D., Campos, A. T., Cruz, V. F., Amaral, P. I., Freitas, L. C. S. R., & Andrade, R. R. Thermal environment in growing and finishing pig facilities of different building typologies. *Journal of Animal Behaviour and Biometeorology*, 2017, *5*(4), 118-123.

4. Presto Åkerfeldt, M., Nihlstrand, J., Neil, M., Lundeheim, N., Andersson, H. K., & Wallenbeck, A. Chicory and red clover silage in diets to finishing pigs—Influence on performance, time budgets and social interactions. *Organic agriculture*, 2019

5. Bracke, M. B. M. RICHPIG: a semantic model to assess enrichment materials for pigs. *Animal Welfare*, 2008, *17*(3), 289-304.

6. Van de Weerd, H. A., Docking, C. M., Day, J. E., Avery, P. J., & Edwards, S. A. A systematic approach towards developing environmental enrichment for pigs. *Applied Animal Behaviour Science*, 2003, *84*(2), 101-118.

7. Huntsberry, M. E., Charles, D., Adams, K. M., & Weed, J. L. The foraging ball as a quick and easy enrichment device for pigs (Sus scrofa). *Lab animal*, 2008, *37*(9), 411.

8. Young, R. J., Carruthers, J., & Lawrence, A. B. The effect of a foraging device (The 'Edinburgh Foodball') on the behaviour of pigs. *Applied Animal Behaviour Science*, 1994, *39*(3-4), 237-247.

9. Beattie, V. E., Walker, N., & Sneddon, I. A. Preference testing of substrates by growing pigs. *Animal Welfare*, 1998, *7*(1), 27-34.

10. Studnitz, M., Jensen, M. B., & Pedersen, L. J. Why do pigs root and in what will they root?: A review on the exploratory behaviour of pigs in relation to environmental enrichment. *Applied animal behaviour science*, 2007, *107*(3-4), 183-197.

11. Lawrence, A. B., Newberry, R. C., & Špinka, M. Positive welfare: What does it add to the debate over pig welfare?. In *Advances in Pig Welfare,* 2018 (pp. 415-444). Woodhead Publishing.

12. Mkwanazi, M. V., Ncobela, C. N., Kanengoni, A. T., & Chimonyo, M. Effects of environmental enrichment on behaviour, physiology and performance of pigs—A review. *Asian-Australasian journal of animal sciences*, 2019, *32*(1), 1.

13. Yang, C. H., Ko, H. L., Salazar, L. C., Llonch, L., Manteca, X., Camerlink, I., & Llonch, P. Pre-weaning environmental enrichment increases piglets' object play behaviour on a large scale commercial pig farm. *Applied Animal Behaviour Science*, 2018, *202*, 7-12.

14. Blackshaw, J. K., Thomas, F. J., & Lee, J. A. The effect of a fixed or free toy on the growth rate and aggressive behaviour of weaned pigs and the influence of hierarchy on initial investigation of the toys. *Applied Animal Behaviour Science*, 1997, *53*(3), 203-212.

15. Scott, K., Taylor, L., Gill, B. P., & Edwards, S. A. Influence of different types of environmental enrichment on the behaviour of finishing pigs in two different housing systems: 3. Hanging toy versus rootable toy of the same material. *Applied Animal Behaviour Science*, 2009, *116*(2-4), 186-190.

16. Apple, J. K., & Craig, J. V. The influence of pen size on toy preference of growing pigs. *Applied Animal Behaviour Science*, 1992, *35*(2), 149-155.

17. Yang, C. H., Ko, H. L., Salazar, L. C., Llonch, L., Manteca, X., Camerlink, I., & Llonch, P. Pre-weaning environmental enrichment increases piglets' object play behaviour on a large scale commercial pig farm. *Applied Animal Behaviour Science*, 2018, *202*, 7-12.

18. Douglas, C., Bateson, M., Walsh, C., Bédué, A., & Edwards, S. A. Environmental enrichment induces optimistic cognitive biases in pigs. *Applied Animal Behaviour Science*, 2012, *139*(1-2), 65-73.

19. Bracke, M. B. Multifactorial testing of enrichment criteria: Pigs 'demand' hygiene and destructibility more than sound. *Applied Animal Behaviour Science*, 2007, *107*(3-4), 218-232.

20. Rius, M. M., Cozzi, A., Bienboire-Frosini, C., Teruel, E., Chabaud, C., Monneret, P., ... & Pageat, P. Selection of putative indicators of positive emotions triggered by object and social play in mini-pigs. *Applied Animal Behaviour Science*, 2018, *202*, 13-19.

21. Bracke, M. B. Chains as proper enrichment for intensively-farmed pigs?. In *Advances in Pig Welfare* 2018 (pp. 167-197). Woodhead Publishing.

22. Tallet, C., Brajon, S., Devillers, N., & Lensink, J. Pig–human interactions: Creating a positive perception of humans to ensure pig welfare. In *Advances in Pig Welfare* 2018 (pp. 381-398). Woodhead Publishing.

23. Baxter, E. M., Lawrence, A. B., & Edwards, S. A. (2011). Alternative farrowing systems: design criteria for farrowing systems based on the biological needs of sows and piglets. *Animal*, *5*(4), 580-600.

24. Jensen, P., & Recén, B. (1989). When to wean—observations from free-ranging domestic pigs. *Applied Animal Behaviour Science*, *23*(1-2), 49-60.

25. Damm, B. I., Moustsen, V., Jørgensen, E., Pedersen, L. J., Heiskanen, T., & Forkman, B. (2006). Sow preferences for walls to lean against when lying down. *Applied Animal Behaviour Science*, *99*(1-2), 53-63.

26. PigSAFE developed by researchers at SRUC and Newcastle University, piglet protection recommendations: https://www.freefarrowing.org/downloads/file/28/piglet_protection_recommendations

27. Wechsler, B., & Weber, R. (2007). Loose farrowing systems: challenges and solutions. *Animal welfare*, *16*(3), 295-307.

28. Büttner, K., Czycholl, I., Basler, H., & Krieter, J. Effects of an intensified human–animal interaction on tail biting in pigs during the rearing period. *The Journal of Agricultural Science*, 2018, *156*(8), 1039-1046.

29. Rault, J. L. Effects of positive and negative human contacts and intranasal oxytocin on cerebrospinal fluid oxytocin. *Psychoneuroendocrinology*, 2016, *69*, 60-66.

30. Tallet, C., Sy, K., Prunier, A., Nowak, R., Boissy, A., & Boivin, X. Behavioural and physiological reactions of piglets to gentle tactile interactions vary according to their previous experience with humans. *Livestock Science*, 2014, *167*, 331-341.

31. Muns, R., Rault, J. L., & Hemsworth, P. Positive human contact on the first day of life alters the piglet's behavioural response to humans and husbandry practices. *Physiology & behavior*, 2015, *151*, 162-167.

32. Verdon, M., & Rault, J. L. (2018). Aggression in group housed sows and fattening pigs. In *Advances in Pig Welfare* (pp. 235-260). Woodhead Publishing.

33. Camerlink, I., Turner, S. P., Farish, M., & Arnott, G. (2019). Advantages of social skills for contest resolution. *Royal Society open science*, *6*(5), 181456.

34. Telkänranta, H., Swan, K., Hirvonen, H., & Valros, A. Chewable materials before weaning reduce tail biting in growing pigs. *Applied Animal Behaviour Science*, 2014, *157*, 14-22.

35. Martin, J. E., Ison, S. H., & Baxter, E. M. The influence of neonatal environment on piglet play behaviour and post-weaning social and cognitive development. *Applied Animal Behaviour Science*, 2015,*163*, 69-79.

36. Tozawa, A., Tanaka, S., & Sato, S. The effects of components of grazing system on welfare of fattening pigs. *Asian-Australasian journal of animal sciences*, 2016, *29*(3), 428.

37. Bracke, M. B., Zonderland, J. J., Lenskens, P., Schouten, W. G., Vermeer, H., Spoolder, H. A., ... & Hopster, H. Formalised review of environmental enrichment for pigs in relation to political decision making. *Applied Animal Behaviour Science*, 2006, *98*(3-4), 165-182.

38. Averós, X., Brossard, L., Dourmad, J. Y., de Greef, K. H., Edge, H. L., Edwards, S. A., & Meunier-Salaün, M. C. A meta-analysis of the combined effect of housing and environmental enrichment characteristics on the behaviour and performance of pigs. *Applied Animal Behaviour Science*, 2010, *127*(3-4), 73-85.

39. Imfeld-Mueller, S., & Hillmann, E. Anticipation of a food ball increases short-term activity levels in growing pigs. *Applied animal behaviour science*, 2012, *137*(1-2), 23-29.

40. Manteuffel, G., Langbein, J., & Puppe, B. From operant learning to cognitive enrichment in farm animal housing: bases and applicability. *Animal Welfare*, 2009, *18*(1), 87-95.

41. De Jonge, F. H., Tilly, S. L., Baars, A. M., & Spruijt, B. M. On the rewarding nature of appetitive feeding behaviour in pigs (Sus scrofa): do domesticated pigs contrafreeload?. *Applied Animal Behaviour Science*, 2008, *114*(3-4), 359-372.

42. Manteuffel, G., Mannewitz, A., Manteuffel, C., Tuchscherer, A., & Schrader, L. Social hierarchy affects the adaption of pregnant sows to a call feeding learning paradigm. *Applied animal behaviour science*, 2010, *128*(1-4), 30-36.

43. Zebunke, M., Puppe, B., & Langbein, J. Effects of cognitive enrichment on behavioural and physiological reactions of pigs. *Physiology & behavior*, 2013, *118*, 70-79.

44. Puppe, B., Ernst, K., Schön, P. C., & Manteuffel, G. Cognitive enrichment affects behavioural reactivity in domestic pigs. *Applied Animal Behaviour Science*, 2007, *105*(1-3), 75-86.

45. Ernst, K., Tuchscherer, M., Kanitz, E., Puppe, B., & Manteuffel, G. Effects of attention and rewarded activity on immune parameters and wound healing in pigs. *Physiology & behavior*, 2006, *89*(3), 448-456.

46. Kalbe, C., & Puppe, B. Long-term cognitive enrichment affects opioid receptor expression in the amygdala of domestic pigs. *Genes, Brain and Behavior*, 2010, *9*(1), 75-83.

47. Zebunke, M., Langbein, J., Manteuffel, G., & Puppe, B. Autonomic reactions indicating positive affect during acoustic reward learning in domestic pigs. *Animal Behaviour*, 2011, *81*(2), 481-489.

48. Turner, S. P., Camerlink, I., Baxter, E. M., D’Eath, R. B., Desire, S., & Roehe, R. Breeding for pig welfare: Opportunities and challenges. In *Advances in Pig Welfare* 2018 (pp. 399-414). Woodhead Publishing.

49. Baxter, E. M., Adeleye, O. O., Jack, M. C., Farish, M., Ison, S. H., & Edwards, S. A.. Achieving optimum performance in a loose-housed farrowing system for sows: the effects of space and temperature. *Applied Animal Behaviour Science*, 2015, *169*, 9-16.

50. Baxter, E. M., Rutherford, K. M. D., d'Eath, R. B., Arnott, G., Turner, S. P., Sandøe, P., ... & Lawrence, A. B. The welfare implications of large litter size in the domestic pig II: management factors. *Animal Welfare*, 2013, *22*(2), 219-238.

51. Rodenburg, T. B., et al. "Breeding amiable animals? Improving farm animal welfare by including social effects in breeding programmes." *Anim Welf* 19.Suppl 1 (2010): 77-82.
