# Supplementary material for: Advancing a “Good Life” for Farm Animals: Development of Resource Tier Frameworks for On-Farm Assessment of Positive Welfare for Beef Cattle, Broiler Chicken and Pigs
Source: Animals (Basel). 2022 Feb 23;12(5):565. doi: 10.3390/ani12050565 (PMC8908822; doi:10.3390/ani12050565)
Supplement: Supplementary file 1 [file animals-12-00565-s001.zip › S4 Beef cattle Good Life Framework post pilot.pdf]

Table S4. Beef cattle Good Life Framework resulting from refinement after the piloting exercise.

**Positive Welfare Opportunity for Comfort** by choice of physical environment	*Cattle should be able to exercise individual preferences for their physical comfort at all times*
+ Are cattle untethered on the farm?	
+ If cattle are housed some or all of the time in sheds with slatted floors, are the slats covered with rubber matting?	
+ For cattle kept solely at pasture, do cattle have access to sheltered dry lying areas?	
++ If cattle have access to housing, are cattle loose housed with free access to an unrestricted/undefined lying area that allows cattle the choice to lie in any orientation and to switch sides easily?	
++ If cattle have access to housing, is all flooring solid? (i.e. no slatted flooring)	
++ If access to housing, is bedding material (e.g. straw) kept deep (compressible), clean and dry i.e.by regular renewal OR if outdoor only, is lying area kept dry and free of poaching?	
+++ Can all cattle lie down simultaneously with room to spare (e.g. at least 10 to 20% spare lying space), to allow cattle choice of where to lie and who to lie next to?	
+++ Is cattle comfort monitored (using any of the following: cameras, activity meters, behaviour observations, welfare outcome scores) and resources /management practices altered to improve comfort where necessary?	
**Positive Welfare Opportunity Comfort** by choice of thermal environment	*Cattle should be able to exercise individual preferences for their thermal comfort at all times*
+ Is the potential for heat stress AND cold stress monitored and actions taken to reduce thermal stress? (e.g. extra water sources in heat, extra bedding in cold)	
+ In housing and/or shelters, is there sufficient dry bedding, renewed regularly?	
++ Can cattle choose to be outdoors for at least part of the day or night, AND can they choose to be indoors or have access to a shelter for at least part of the day or night?	
++ Is there shelter available for at least some animals e.g. hedges, trees, sheds, walls, portable structures?	
+++ Is sufficient shelter provided for all animals to stand or lie in the shelter and shade at the same time?	
+++ For cattle both indoors and outdoors, do cattle of all ages have a choice of temperatures (e.g. nearer to or further from open or ventilated areas in housing, access to windbreaks/shelters outdoors, shade of different intensities i.e. partial or full shade) and can they always avoid thermal extremes (e.g. fans and ventilation for heat in housing, extra bedding and no excessive draughts in cold weather both indoors and outdoors)?	
**Positive Welfare Opportunity Pleasure** by play	*Cattle should be able to exercise individual preferences for play*
++ Do cattle of all ages AT SOME TIMES have enough space, appropriate design of housing/pasture (layout and non-slip floor surface) to allow some opportunities for play including running/jumping/chasing and playing with other cattle (social play behaviour)?	

+++ Do cattle of all ages AT ALL TIMES have the opportunity to run, jump, chase and play with other cattle at all times? Excluding calving, hospitalisation due to disease.	
**Positive Welfare Opportunity Pleasure** by breeding and nurturing experiences	*Cattle should be able to have positive reproductive and nurturing experiences*
+ IF natural weaning is not carried out, are calves kept with their dam until at least 45 days old?	
+ IF natural weaning is not carried out, are there any interventions in place to protect against the stress of nutritional weaning (stopping access to milk) AND social weaning (stopping access to mother)? Examples include: solid food offered to calves from a few days of age (this includes access to grass); fence line separation of calves and dams during separation; calves kept in stable groups of familiar individuals in a familiar environment after separation from the dam	
++ IF natural weaning is not carried out, are calves kept with their dam until they are at least 3 months old?	
++ IF natural weaning is not carried out, is gradual weaning (both nutritional and social) carried out? Examples include two step weaning, step one of suckling prevention (anti suckling devices, restricted suckling periods), step two fence line separation to allow physical and social contact?	
+++ Are calves kept with their own dam until natural weaning (i.e. until at least 8 months of age)?	
**Positive Welfare Opportunity Confidence** by positive experience with stock keepers	*Cattle should be able to have positive experiences of people when encountered*
+ NO goads (i.e. an object specifically to hit cattle) are used when handling cattle - answer YES if this is true. (This does not include a stick that is held but not used to hit cattle).	
+ Does the design and layout if handling systems encourage natural, safe movement of animals (for example suitable race with wider funnel at the start, sweeping gate behind, curves in the race, boarded sides) AND/OR animals are made very familiar with the handling system and exhibit low stress when handled?	
++ Do stock persons spend time regularly in the presence of cattle of all ages?	
++ Are young cattle trained (for example, using positive handling, attention, food) from a young age to move around calmly (when led, driven or being examined)?	
+++ Do stock persons spend time talking to and positively interacting with cattle e.g. scratching heads/tails?	
+++ Do stock keepers undergo any type of training on cattle welfare e.g. practical welfare techniques, understanding cattle behaviour and good handling practices, developing positive attitudes towards cattle? (Training counts as anything that can be logged/evidenced)	
**Positive Welfare Opportunity Confidence** by positive social experiences within the herd	*Cows' confidence should be promoted by the opportunity to have positive social experiences within the herd, including establishment and maintenance of a stable social hierarchy and being able to learn about their environment from other cattle.*

+ Are cattle kept in such a way that they can maintain small, stable social groups of familiar individuals, no more than 70 animals to enable individual recognition?	
++ Are calves/youngstock kept and reared together from the time they are separated from their dams or arrive at the farm?	
+++ If adult cattle are present, does a bull run with the herd, some or all the time (including sweeper bull / vasectomised bull) allowing natural behaviour to be expressed and natural social hierarchy and herd stability to be maintained?	
+++ Are breeding bulls isolated only for biosecurity/ disease screening and at all other times kept with or in sight of other stock, providing safety can be maintained?	
**Positive Welfare Opportunity Interest** by a positively enriched environment	*Cattle should have opportunities to explore and play in an enriched environment throughout their lives*
+ Is there a choice between 2 or more positive activities for exploration? (e.g. INDOORS hay balls, cardboard boxes, automatic or manual brushes, padded posts, sand pit, hay bales, OUTDOORS tree stumps, hedges and other vegetation, tractor tyres)?	
++ Are there opportunities for choice between two (or more) positively stimulating activities (see examples in welfare +) provided CONTINUOUSLY throughout life?	
+++ Are there opportunities for choice between two (or more) positively stimulating activities, provided continuously and CHANGED or replenished frequently throughout life? (i.e. between every 2 weeks and 3 months). This INCLUDES changing fields/giving cattle a choice of fields when outside.	
**Positive Welfare Opportunity Interest** by pasture choices	*Interest should be promoted by the opportunity to be able to exercise individual preferences about their access to and use of pasture*
+ Do cattle have access to pasture during parts of the day/night during the summer grazing season?	
+ In low rainfall locations i.e. less than 28 inches annual rainfall, free draining soil types, do all cattle have access to straw corrals with a pad of well drained bedding (e.g. woodchip) during parts of the day and night?	
++ Do all cattle have choice to access pasture at all times of day/night during the summer grazing season?	
+++ Do cattle have easy access to sheltered pasture (trees, hedges, artificial shelter) at all times of day/night across an extended grazing season i.e. at least 180-200 days a year?	
+++ Are both access and shelter sufficient to allow pasture to be safely used in winter, with efforts to minimise mud patches/ poaching of high traffic areas?	
**Positive Welfare Opportunity Pleasure by food enrichment**	*Cattle should be able to exercise individual preferences for type of food and how it is obtained*
+ Are cattle offered, from weaning, sufficient amount of nutritionally balanced diet consisting of fibrous/ forage rather than high concentrate feed?	
+ IF housed, are cattle provided with long fibre, such as straw or hay, to allow exploratory behaviour with their mouths e.g. manipulation, licking, chewing, ruminating?	

++ Are cattle provided with some variety in their diet? i.e. When INDOORS, at least two types of forage (grass silage, maize silage, hay, whole crop silage, straw, turnips or fodder beet, bales), when OUTDOORS, some variety in the ley i.e. not just ryegrass?	
+++ Are cattle offered a choice of pasture herbage i.e. diverse leys outdoors AND access to trees/hedges to browse?	
**Positive welfare Opportunity Healthy Life** by management policy for positive health	*Stock persons should manage day to day cattle health effectively*
+ Is a health and welfare plan implemented and reviewed frequently i.e. every 6 months or more often and corrective action taken?	
+ Are medicines used as required, in conjunction with good management, rather than routinely or blanket approach basis?	
++ Do stock keepers know the individual habits and preferences of most animals they manage?	
++ If a stock keeper recognises changes in cattle behaviour that suggest poor health/welfare, do they act on this within 24 hours to investigate and rectify the problem?	
+++ Is there regular dialogue with the vet AND/OR scheme welfare advisor if applicable?	
+++ Do farm staff take part in welfare improvement activities with wider benefits? i.e. member of welfare policy/advisory group, peer advisor, welfare discussion groups, participates in on-farm welfare research/innovation.	
**Positive welfare Opportunity Healthy Life by positive genetic selection for long term health and welfare**	*Stock keepers should positively influence the long-term health and welfare of cattle*
+ When selecting or breeding own replacement animals (heifers and/or bulls) or selecting sires, are undesirable traits recognised and avoided, to improve herd health and welfare? e.g. sire/breed calving ease (to avoid caesareans), calf vigour, pelvic measurements and calm temperament.	
+ If own replacements are not home bred (store cattle, heifers, cows, bulls, AI sires) is feedback given to the rearers/ breeders/ breed societies/ AI companies as to what traits are important when purchasing livestock or AI straws?	
++ Are breeding choices for future herd health and welfare (e.g. avoidance of double muscling) valued equally to breeding choices made for growth rates and other production factors?	
++ If surrogate dams are used as recipients for embryos, the breed/breed mix, calving ease scores of the resulting calves is not likely to result in caesarean births?	
+++ When selecting replacements are choices made for future long-term improvement of herd health and welfare and resilience valued more than breeding choices made for growth rate and other production factors?	
+++ Is feedback provided to the relevant party (e.g. breeding companies/breed societies) to improve genetic evaluations and provide other beef farmers the opportunity to select cattle best suited to their own production system/ resources?	
**Positive welfare Opportunity Healthy Life** by promoting a natural body type	*Cattle should be able to live a life without changing their nature.*
+ No dehorning (removal of a grown horn) is undertaken - answer YES if this is true	

+ No branding used as a method of identification - answer YES if this is true
+ If castration and/or disbudding (removal of horn buds) is undertaken is anaesthetic AND long-term pain relief used for all ages of cattle including calves less than 2 months old?
+ If castration is carried out, is it only done at a young age i.e. calves not adults – is this true?
+ Rubber ring or bloodless (burdizzo) castration is not carried out - answer YES if this is true
++ No disbudding (removal of horn bud) is carried out - answer YES if this is true
+++ Neither dehorning/disbudding nor castration is carried out - answer YES if this is true
