# Supplementary material for: Advancing a “Good Life” for Farm Animals: Development of Resource Tier Frameworks for On-Farm Assessment of Positive Welfare for Beef Cattle, Broiler Chicken and Pigs
Source: Animals (Basel). 2022 Feb 23;12(5):565. doi: 10.3390/ani12050565 (PMC8908822; doi:10.3390/ani12050565)
Supplement: Supplementary file 1 [file animals-12-00565-s001.zip › S5 Broiler Good Life Framework post pilot.pdf]

Table S5. Broiler chicken Good Life Framework resulting from refinement after the piloting exercise.

**Positive Welfare Opportunity for Comfort** by choice of physical environment	*Birds should be able to exercise individual preferences for their physical comfort at all times*
+ Are birds given elevated resting areas in the form of flat raised platforms?	
++ Do elevated platforms have ramps for access?	
++ Is there a provision of perches AND use of slow growing strain/breed that are better able to utilise perches?	
+++ Is there sufficient perch space for all birds to have choice to rest elevated and undisturbed?	
+++ Is there suitable litter to a depth of > 10cm?	
+++ Are birds given a choice or 2 or more types of suitable litter? (e.g. wood- based litter, peat substitute, straw, sand or equivalent)	
**Positive Welfare Opportunity Comfort** by choice of thermal environment	*Birds should be able to exercise individual preferences for their thermal comfort at all times*
+ Is there protection from draughts in resting/perching area?	
+ Is there access to perches/platforms to improve opportunities for thermoregulation?	
+ Is roof insulation used?	
++ Is there access to outdoors to add a choice of temperatures?	
++ Is there shade and windbreaks throughout the range as protection from the weather?	
+++ Are there pop hole rooves and/or cover from wind and rain outside NEAR to pop holes e.g. veranda, shelters?	
**Positive Welfare Opportunity Pleasure** by food enrichment	*Birds should be able to exercise individual preferences for food and how it is obtained*
+ Is any food offered outside of standard feeders? E.g. scatter fed, offered in foraging device (such as pecking block, hanging object such as wire bird suet feeder filled with food, strung up vegetable, maize cobs)?	
++ Are wholegrain and/or insects scatter fed?	
+++ Are forage crops available? (e.g. brassicas, grass, clover, peas, vetch, lupins, quinoa)	
+++ Are puzzle feeders (e.g. food ball) provided to prolong provision of food enrichment?	
**Positive Welfare Opportunity Pleasure** by play	*Birds should be able to able to exercise individual preferences for play*

++ Are slow growing strains/breeds used, so that birds are more capable of feeling active and therefore carrying out play behaviour?	
+++Is sufficient space (stocking density maximum of 30kg/m2) provided throughout the growing period to allow birds to perform play behaviours such as sparring (play-fight), jumping, running and wing flapping?	
+++ Are novel objects provided to encourage play behaviour e.g. hanging items, forage blocks/bales?	
+++ Is there a policy for monitoring and acting on play behaviour e.g. providing objects and/or more space to encourage and increase play behaviour?	
**Positive Welfare Opportunity Pleasure by breeding and nurturing experiences**	*Birds should be able to have positive reproductive and nurturing experiences*
+ Are dark brooders used for chicks?	
++ Is there a provision of adult hens for chicks?	
+++ Are broiler chicks allowed to imprint on adult hens?	
**Positive Welfare Opportunity Confidence** by positive experience with stock keepers	*Birds should be able to have positive experiences of people when encountered*
+ Are stock keepers calm (e.g. slow movements) and quiet around the birds?	
+ Is handling gentle, with birds caught and handled in an upright position?	
++ Is thinning prohibited?	
++ Is there a promotion of positive human - animal relationship by associating presence of humans with non-aversive interactions e.g. talking to birds, maintaining regular visual contact, gentle touching, as well as positive interactions e.g. feeding from hand, whenever in the presence of birds doing routine tasks e.g. feeding and cleaning?	
+++ Do stock keepers undergo any type of training on broiler welfare e.g. practical welfare techniques, understanding chicken behaviour and good handling practices, developing positive attitudes towards chickens, understanding of chicken sensitivity to negative behaviours, e.g. low stress stock handling poultry passport? (Training counts as anything that can be logged/evidenced)	
+++ Are there efforts to improve the predictability /controllability for birds by signalling stressful events e.g. knocking on the door before entering?	
**Positive Welfare Opportunity Confidence** by positive social interactions	*Birds should be able to have positive social experiences withing their group*

+ Are all resources (food, water, enrichment) spread out evenly to avoid competition between birds?	
+ Does stocking rate (maximum 30kg/m2) allow space to enable birds to avoid antagonistic interactions and choose who to be close to?	
++Are there visual barriers (e.g. bales of plastic wrapped shavings) vertical panels and/or raised platforms to allow birds to avoid negative social interactions and create the perception of smaller group sizes?	
+++ Are there small flock sizes to enable individual recognition and a stable hierarchy?	
**Positive Welfare Opportunity Interest** by a positively enriched environment	*Birds should have opportunities to explore and play in an enriched environment throughout their lives*
+ Is there an even distribution of at least 1 type of foraging item (breeze blocks, forage/lucerne blocks, chopped carrots, nets with cut straw/hay, bale of wood shavings) and 1 manipulation item (e.g. hanging items, CDs, stationary bunches of string/baler twine, spherical objects)?	
++ Are foraging and manipulation items changed on a regular basis within the same crop to maintain novelty/interest?	
++ Is there reduced stocking density (maximum 30kg/m2) to allow for increased opportunity to forage?	
+++ Are 2 or more types of foraging substrate provided? (e.g. wood-based litter, peat substitute, straw, sand, oat hulls/husks, soil)	
+++ Are there vertical panels and/or barriers (e.g. wooden beams, wood shaving/straw bales) to increase activity, space usage and allow perching and resting?	
+++ Are birds provided with outdoor access, in combination with the use of slow growing strains/breeds?	
+++ Do birds have access to natural structures on the range? (e.g. log piles, fallen down trees on the range, branches, hay bales)	
**Positive Welfare Opportunity Interest by enhanced learning opportunities**	*Birds should be able to experience positive emotional states through cognitive enrichment*
+ Is the complexity of the environment increased to stimulate learning e.g. provision of straw bales, different types of vegetation on the range?	
++ Is daily access (at least 4 hours per day) to range given? (In combination with use of slow growing strains/breeds)	
+++ Is daily access given to learning enrichments e.g. mazes, branches, even distribution of feeding devices and tasks such as puzzle feeders e.g. food ball? (Only in combination with use of slow growing strains/breeds)	
**Positive welfare Opportunity Interest by promoting ranging**	*Birds should be able to have positive experiences of the outdoor environment*

+ Is daily access given to a well-drained range with covered structures e.g. shade and shelter panels and hedges/shrubs?	
+ Do covered structures and hedges/shrubs start no further than 3m from the pop holes AND are distributed evenly throughout the range?	
+ Are slow growing strains/breeds used?	
+++ Are there covered dustbathing opportunities e.g. roofed sandpit distributed evenly on the range?	
+++ Are there other animals (ruminants) using the range?	
+++ Is there a substantial woodland/forest area (at least 25% of range area) for ranging including some mature trees (>2 years)?	
+++ Is there access to edible vegetation e.g. grass, clover, chicory, either scattered or growing on the range?	
**Positive welfare Opportunity Healthy Life** by dustbathing choices	*Birds should be able to exercise individual preferences for dustbathing substrate and location*
+ Is stocking density sufficient to allow space for dustbathing (minimum 30kg/m2)?	
+ Is there continuous access to areas containing dustbathing substrate (e.g. wood-based litter, peat substitute, straw, sand, oat hulls/husks or equivalent)?	
++ Are slow growing strains/breeds used?	
++ Is there access to covered dustbathing areas (e.g. roofed sandpit) outdoors or in a veranda?	
+++ Is there a choice of more than 1 dustbathing substrates in outdoor shelter/veranda?	
+++ Is the litter friable and deeper than 10cm?	
**Positive welfare Opportunity Healthy Life** by resting choices	*Birds should have opportunities to rest undisturbed*
+ Do lighting patterns allow synchronised behaviour (undisturbed rest) including a minimum 6 hours uninterrupted dark?	
+ Is natural light provided? i.e. at least 2 lux covering 3% of the floor	
+ Is litter maintained in a dry and friable condition?	
++ Are perches and/or platforms provided to reduce disturbance and so allow undisturbed rest?	
+++ Are panels and/or barriers (e.g. wooden beams, straw or wood shaving bales) provided to reduce disturbances and so allow undisturbed rest?	

+++ Are birds given the choice of when to rest and when to be active if artificial lighting is used, by using ''dual light intensity choice'' e.g. providing a light intensity of 2 lux in one area/compartment of the shed and 20 lux in another?	
**Positive welfare Opportunity Healthy Life** by management policy for positive health	*Stock keepers should manage day to day health effectively*
+Is a health and welfare programme implemented and reviewed frequently plus action taken to reduce and alleviate the cause of any health and welfare problems?	
+ Medicines other than vaccines are NOT routinely used - answer YES if this is true	
+ Is there a policy for monitoring and culling sick birds?	
++ IF birds have outdoor access, are procedures in place to reduce risk of disease in outdoor range as well as predation e.g. access to cover?	
++ Is thinning prohibited?	
++Is there regular dialogue (at least once per crop) with vet and/or scheme welfare advisor?	
+++ Do farm staff take active part in welfare activities with wider benefits (e.g. member of scheme policy/management group, peer advisor, on farm welfare research)?	
**Positive welfare Opportunity Healthy Life** by positive genetic selection for long term health and welfare	*Stock keepers should influence long term health and welfare*
+ Does the farm manager recognise undesirable side effects of genetic selection for production efficiency and choose replacement birds to reduce/mitigate for current health and welfare problems within flock (e.g. lameness, sudden death syndrome, ascites) i.e. choose slow growing strains/breeds over fast growing ones where manager has control over choice of replacements?	
+ If own replacements are not selected/bred by the unit manager, is feedback given to the breeders/genetic companies/hatcheries which supply the chicks, as to what traits are important to the manager?	
++ Are breed/bird choices made to mitigate potential issues for future flock health and welfare valued equally to choices made for growth rate and other production factors?	
+++ Are replacements chosen for long term improvement of flock health and welfare, resilience and metabolic normality, valuing these more than choices made for growth rate and other production factors?	
