# Supplementary material for: Advancing a “Good Life” for Farm Animals: Development of Resource Tier Frameworks for On-Farm Assessment of Positive Welfare for Beef Cattle, Broiler Chicken and Pigs
Source: Animals (Basel). 2022 Feb 23;12(5):565. doi: 10.3390/ani12050565 (PMC8908822; doi:10.3390/ani12050565)
Supplement: Supplementary file 1 [file animals-12-00565-s001.zip › S6 Pig Good Life Framework post pilot.pdf]

Table S6. Pig Good Life Framework resulting from refinement after the piloting exercise.

**Positive Welfare Opportunity for Comfort** by choice of physical environment	*Animals should be able to exercise individual preferences for their physical comfort at all times*
+ Is the lying area of solid construction (including solid earth outdoors)?	
+ Is bedding to a depth of >2cm provided?	
++ Is bedding to a depth of >10cm?	
+++ Is there more space than needed for all pigs to lie down simultaneously, to allow choice of where to lie?	
**Positive Welfare Opportunity Comfort** by choice of thermal environment	*Animals should be able to exercise individual preferences for their thermal comfort at all times*
+ Do pigs have a choice of temperatures? e.g. shelter/shade outdoors, access to open air (open-sided barn), area of deeper bedding, area of bare concrete floor	
+ Is there enough space for pigs to choose which thermal environment to be in?	
++ Do pigs have the opportunity to access a (non-faecal) wallow? (Outdoors and/or indoors e.g. water pit)?	
+++ Do pigs have the opportunity to spend at least part of the day/night outdoors with access to shade?	
+++ Is there enough space in wallows for all pigs to use at the same time?	
+++ Is there enough space for all pigs to access shade at the same time?	
**Positive Welfare Opportunity Pleasure** by food enrichment.	*Animals should be able to exercise individual preferences for type of food and how it is obtained*
+ Do pigs receive roughage such as silage, available clean each day?	
+ Do pigs receive part of their diet scattered widely on a clean floor daily?	
++ Do pigs receive part of diet scattered widely on rootable substrate daily?	
+++ Do pigs receive other forms of ingestible enrichment e.g. peanuts, hay, straw, vegetables in box, hanging vegetables?	
**Positive Welfare Opportunity Pleasure** by play	*Animals should be able to exercise individual preferences for play*
+ is there at least one clean toy designed to induce object play (e.g. soft malleable rubber toys and balls, ropes, plastic piping) OR enough space to allow running at least 10 pig paces in one direction to allow for social and locomotor play?	
++ is there at least one clean toy designed to induce object play (e.g. soft malleable rubber toys and balls, ropes, plastic piping) AND enough space to allow running at least 10 pig paces in one direction to allow for social and locomotor play?	

+++ Is there more than one clean toy? (e.g. soft malleable rubber toys and balls, ropes, plastic piping)	
+++ Are toys changed on a regular basis so that the same toy is not presented within at least a one-month period?	
**Positive Welfare Opportunity Pleasure** by breeding and nurturing experiences	*Animals should be able to have positive reproductive and nurturing experiences*
+ Is enough space provided for the sow to turn around during the farrowing period?	
+ Is nesting material (e.g. long-stemmed straw) provided 48 hours to farrowing?	
+ Do piglets have access to the sow for 33 - 39 days?	
+ Is weaning gradual? E.g. social separation from the sow in increasing duration and frequency over time?	
++ Is the nesting area enclosed by three solid dark walls?	
++ Does the nesting area allow for gradual lying down?	
++ Is there sufficient nesting material to build and replenish a nest approximately 2m in diameter?	
++ Is there sufficient space for the sow to be able to move away from the piglets?	
++ Do piglets have access to the sow for 40 - 55 days?	
+++ Is there enough space for a nesting area and an activity area for the sow?	
+++ Do the piglets have access to the sow for 56 days or more?	
**Positive Welfare Opportunity Confidence** by positive experience with stockpersons	*Animals should be able to have positive experiences of people when encountered*
+ NO goads (i.e. an object specifically to hit pigs) are used when handling pigs - answer YES if this is true. (This does not include a stick that is held but not used to hit pigs).	
+ Is calm speech used around the pigs at all times (no shouting)?	
+ Are all handling procedures carried out gently?	
++ Is the presence of humans associated with positive events such as offering food, positive interactions such as stroking and scratching, to at least some of the pigs whenever carrying out routine tasks e.g. feeding?	
+++ Is time dedicated to positive interactions (gentle stroking/scratching/offering food) with all pigs/piglets, on a regular basis?	
+++ Do stock keepers undergo any type of training on pig welfare e.g. practical welfare techniques, understanding pig behaviour and good handling practices, developing positive attitudes towards pigs (Training counts as anything that can be logged/evidenced)	

<table>
<tr><td>Positive Welfare Opportunity Confidence by positive social interactions</td><td>Animals should be able to have positive social experiences within their group</td></tr>
<tr><td colspan="2">+ Are strategies in place to reduce negative interactions between pigs? Examples include: number and layout of resources avoid competition; layout of pen and visual barriers allow escape/prevent cornering; if pigs are mixed, group sizes are large (>20 pigs); extra enrichment provided at point of mixing; pigs are mixed into groups of same sex and with obviously different weights)</td></tr>
<tr><td colspan="2">++ Is there ample space to allow pigs to choose who to spend time with and who to avoid?</td></tr>
<tr><td colspan="2">++ Are piglets socialised during lactation period (co-mingle with other piglets) after 10 days of age (to improve social skills)?</td></tr>
<tr><td colspan="2">+++ Is any mixing done before weaning, so that pigs are kept in stable groups of familiar individuals for the rest of their life?</td></tr>
<tr><td>Positive Welfare Opportunity Interest by a positively enriched environment</td><td>Animals should have opportunities to explore and play in an enriched environment throughout their lives</td></tr>
<tr><td colspan="2">+ Do pigs have access to at least 1 rooting substrate? Substrates to allow pigs to root, investigate, chew and play include: straw, a peat substitute, mushroom compost (or other type of compost), soil, sand, sawdust, wood shavings, wood bark, hay, grass.</td></tr>
<tr><td colspan="2">++ Do pigs have access to 1 rooting substrate AND 1 item of CLEAN object enrichment. Object enrichment includes hanging objects e.g. hanging branched chain, soft malleable rubber dog toy, rope, plastic ball, plastic piping, soft wood or logs, and non-hanging items e.g. sticks, cardboard boxes, branches, loose logs, wooden planks.</td></tr>
<tr><td colspan="2">+++ Is object enrichment changed to different objects on a regular basis so that the same object/toy is not presented within at least a one-month period?</td></tr>
<tr><td colspan="2">+++ Is object enrichment made of chewable and destructible materials such as ropes, paper and jute AND/OR is object enrichment compound in the form of mixed substrates e.g. long straw with fir branches, straw with forest bark and branches, or objects made of a combination of materials, e.g. ropes and rubber hoses, and is it destructible, deformable, complex, changeable and sometimes containing ingestible parts?</td></tr>
<tr><td colspan="2">+++ Is access to outdoor pasture or woodland or root crops provided during daytime?</td></tr>
<tr><td>Positive Welfare Opportunity Interest by enhanced learning opportunities</td><td>Animals should be able to experience positive emotional states through cognitive enrichment</td></tr>
<tr><td colspan="2">+ Is there some form of challenge in finding food e.g. food scattered/hidden?</td></tr>
<tr><td colspan="2">++ Is some food provided in ‘puzzle feeders’ such as food ball? (With enough puzzle feeders provided to avoid competition?)</td></tr>
</table>

+++ Are pigs given access to a complex environment outdoors (i.e. range of different vegetation types/sizes) AND/OR any type of novelty in their environment on a regular basis (e.g. changes in enrichment, move to new paddocks) to drive learning about their environment?	
+++ Are ‘puzzle feeders’ of increasing difficulty (requiring more manipulation to release food) provided, to maintain cognitive challenge?	
**Positive Welfare Opportunity Healthy Life** by management policy for positive health	*Animal carers should manage day-to-day animal health effectively*
+ Is the health and welfare programme implemented and reviewed frequently i.e. every 6 months or more often, plus action taken to reduce or alleviate the cause of any health and welfare problems?	
+ Medicines other than vaccines are NOT routinely used - answer YES if this is true	
+ Do stockkeepers manage vulnerable groups (e.g. piglets) with attention and positive management strategies for their group needs?	
++ Is there regular dialogue with a vet and/or a scheme welfare advisor?	
+++ Do stockkeepers manage vulnerable individual animals (e.g. piglets) with attention and positive management strategies for their individual needs?	
+++ Do stockkeepers take active part in welfare activities with wider benefits (e.g. member of scheme policy/ management group, peer advisor, on-farm welfare research)?	
**Positive welfare Opportunity Healthy Life** by positive genetic selection for long-term health and welfare	*Animal carers should positively influence the long-term health and welfare of animals*
+ Does the farm manager recognise undesirable side-effects of genetic selection for production efficiency and choose replacement animals to reduce/mitigate for current health and welfare problems within the herd (e.g. piglet mortality, tail biting), where the farm manager has control over choice of replacement animals?	
+ if own replacements are not home bred or selected by the unit manager is feedback given to the breeders/ genetic companies/ rearers as to what traits are important to the manager?	

++ Does the farm manager make choices for potential future health and welfare issues within the herd, valuing these equally to litter size, growth rate and other production factors?	
+++ Are replacement animals chosen for long-term improvement of herd health and welfare, resilience and metabolic normality, valuing these over litter size, growth rate and other production factors? Specific examples include selecting for piglet survival instead of number born, for example by selecting for improved placental efficiency, reduced intra-litter birth weight variability, more robust piglets, and improved maternal behaviour, sows selected for specific loose-farrowing traits, smaller litter size or at least ceasing to select for increased litter sizes, and breeding for improved social relationships.	
**Positive welfare Opportunity Healthy Life** by promoting a natural body type	*Animals should be able to live a life without changing their nature*
+ NOT ALL outdoor sows have nose rings - answer YES if this is true	
+ If a pig is nose-ringed, this ring is NOT replaced if it falls out - answer YES if this is true	
+ NOT ALL pigs and piglets have docked tails - answer YES if this is true	
+ There have been NO tail-biting outbreaks in the past 3 months that have resulted in treatment or euthanasia - answer YES if this is true	
+ NOT ALL piglets have their teeth reduced - answer YES if this is true	
++ THE MAJORITY of sows DO NOT have nose rings - answer YES if this is true	
++ THE MAJORITY of pigs and piglets are NOT tail docked - answer YES if this is true	
++ Are long- and short-tailed pigs are kept in separate groups?	
++ THE MAJORITY of piglets DO NOT have their teeth reduced - answer YES if this is true	
++ IF boars are castrated, is chemical castration used?	
+++ NO sows have nose rings - answer YES if this is true	
+++ NO pigs and piglets tail docked - answer YES if this is true	
+++ NO piglets have had teeth reduced - answer YES if this is true	
+++ NO boars are castrated - answer YES if this is true	
